# Head-to-head comparison of DFO* and DFO chelators: selection of the best candidate for clinical ^89^Zr-immuno-PET

**DOI:** 10.1007/s00259-020-05002-7

**Published:** 2020-09-05

**Authors:** Marion Chomet, Maxime Schreurs, Maria J. Bolijn, Mariska Verlaan, Wissam Beaino, Kari Brown, Alex J. Poot, Albert D. Windhorst, Herman Gill, Jan Marik, Simon Williams, Joseph Cowell, Gilles Gasser, Thomas L. Mindt, Guus A. M. S van Dongen, Danielle J. Vugts

**Affiliations:** 1 Radiology & Nuclear Medicine, Cancer Center Amsterdam, Amsterdam UMC, Vrije Universiteit Amsterdam, De Boelelaan, 1117 Amsterdam, The Netherlands; 2 Genentech Inc., 1 DNA Way, South San Francisco, CA 94080 USA; 3 Institute of Chemistry for Life and Health Sciences, Laboratory for Inorganic Chemical Biology, Chimie ParisTech, PSL University, CNRS, Paris, France; 4 Ludwig Boltzmann Institute for Applied Diagnostics, General Hospital Vienna (AKH), Vienna, Austria

**Keywords:** Bone metastasis model, DFO, DFO*, DFOSq, DFO*Sq, ^89^Zr-immuno-PET

## Abstract

**Purpose:**

Almost all radiolabellings of antibodies with ^89^Zr currently employ the hexadentate chelator desferrioxamine (DFO). However, DFO can lead to unwanted uptake of ^89^Zr in bones due to instability of the resulting metal complex. DFO*-NCS and the squaramide ester of DFO, DFOSq, are novel analogues that gave more stable ^89^Zr complexes than DFO in pilot experiments. Here, we directly compare these linker-chelator systems to identify optimal immuno-PET reagents.

**Methods:**

Cetuximab, trastuzumab and B12 (non-binding control antibody) were labelled with ^89^Zr via DFO*-NCS, DFOSq, DFO-NCS or DFO*Sq. Stability in vitro was compared at 37 °C in serum (7 days), in formulation solution (24 h ± chelator challenges) and in vivo with N87 and A431 tumour-bearing mice. Finally, to demonstrate the practical benefit of more stable complexation for the accurate detection of bone metastases, [^89^Zr]Zr-DFO*-NCS and [^89^Zr]Zr-DFO-NCS-labelled trastuzumab and B12 were evaluated in a bone metastasis mouse model where BT-474 breast cancer cells were injected intratibially.

**Results:**

[^89^Zr]Zr-DFO*-NCS-trastuzumab and [^89^Zr]Zr-DFO*Sq-trastuzumab showed excellent stability in vitro, superior to their [^89^Zr]Zr-DFO counterparts under all conditions. While tumour uptake was similar for all conjugates, bone uptake was lower for DFO* conjugates. Lower bone uptake for DFO* conjugates was confirmed using a second xenograft model: A431 combined with cetuximab. Finally, in the intratibial BT-474 bone metastasis model, the DFO* conjugates provided superior detection of tumour-specific signal over the DFO conjugates.

**Conclusion:**

DFO*-mAb conjugates provide lower bone uptake than their DFO analogues; thus, DFO* is a superior candidate for preclinical and clinical ^89^Zr-immuno-PET.

**Electronic supplementary material:**

The online version of this article (10.1007/s00259-020-05002-7) contains supplementary material, which is available to authorized users.

## Introduction

Positron emission tomography with ^89^Zr-labelled antibodies (^89^Zr-immuno-PET) is a valuable tool to characterise the in vivo behaviour of monoclonal antibodies (mAbs) as well as other drugs with slow clearance from the blood, such as other types of proteins, mAb conjugates, nanoparticles and cells. In this way, immuno-PET can be used to (i) assess target expression; (ii) evaluate the in vivo behaviour of the drug in relation to efficacy and toxicity; (iii) optimise dose, route and schedule of administration; (iv) optimise drug design and (v) select patients with the highest chance of benefiting from drug treatment [1–3]. Over the last decade, the number of clinical studies using ^89^Zr-labelled mAbs has increased enormously, confirming the importance of immuno-PET imaging, especially in oncology [4]. With a half-life of 78.4 h, ^89^Zr is well suited for studying the kinetics of molecules with relatively long plasma half-lives [2, 5]. Currently, the majority of ^89^Zr-immuno-PET studies employ desferrioxamine B (DFO). DFO has been administered to thousands of patients, in free form for the treatment of iron overload or attached to slow kinetic drugs as a chelator for ^89^Zr labelling. However, preclinical studies revealed that the hexadentate ^89^Zr-DFO complex is prone to dissociation in vivo with free ^89^Zr^4+^ predominantly accumulating in bones and joints because of its high affinity for strong electronegative donor atoms such as oxygen and phosphorus in hydroxyapatite present in components of the bones [6–12]. Although the deposition of ^89^Zr in bone has not been routinely observed in the published clinical studies to date, a systematic review is required to further evaluate this question. A resolution to this problem is particularly important since non-specific bone uptake will provide an increased radiation burden to the patient and may contribute to the misidentification of bone metastases. These challenges have prompted the development of a variety of octadentate chelators for ^89^Zr that should result in increased stability of the ^89^Zr complexes [13–19]. Two proprietary chelators, DFO*-NCS [20] and DFOSq [21], both derivatives of DFO, showed preliminary improvements in performance in vitro and in vivo compared with DFO and are currently under consideration for clinical use.

The synthesis of DFO* was first reported by Patra et al. [22] in 2014, and aimed to extend DFO with a fourth hydroxamic acid group. This derivative of DFO presents the advantage of using the oxygen-rich hydroxamate functional group in order to fully coordinate Zr^4+^ [7]. Thus, in 2017, Vugts et al. [23] introduced the bifunctional chelator DFO*-NCS and compared [^89^Zr]Zr-DFO*-NCS-trastuzumab and [^89^Zr]Zr-DFO-NCS-trastuzumab in vitro and performed a first in vivo pilot study in N87-tumour-bearing nude mice. [^89^Zr]Zr-DFO*-NCS-trastuzumab presented promising properties compared with its DFO counterpart with strikingly lower accumulation in bones. In 2016, in parallel to the development of DFO*-NCS, Rudd et al. [24] introduced the bifunctional chelator DFOSq and explained the increased stability of the ^89^Zr-chelator complex by the dione oxygen of the squaramide moiety contributing to a putative octadentate coordination of ^89^Zr. In vitro, [^89^Zr]Zr-DFOSq-Taur was more stable than [^89^Zr]Zr-DFO-*p*-Ph-SO_3_H when challenged with EDTA. In vivo PET imaging and ex vivo biodistribution studies with [^89^Zr]Zr-DFOSq-trastuzumab also revealed reduced liver and bone uptake compared with [^89^Zr]Zr-DFO-NCS-trastuzumab as well as satisfactory HER2 tumour targeting.

**Fig. 1:**
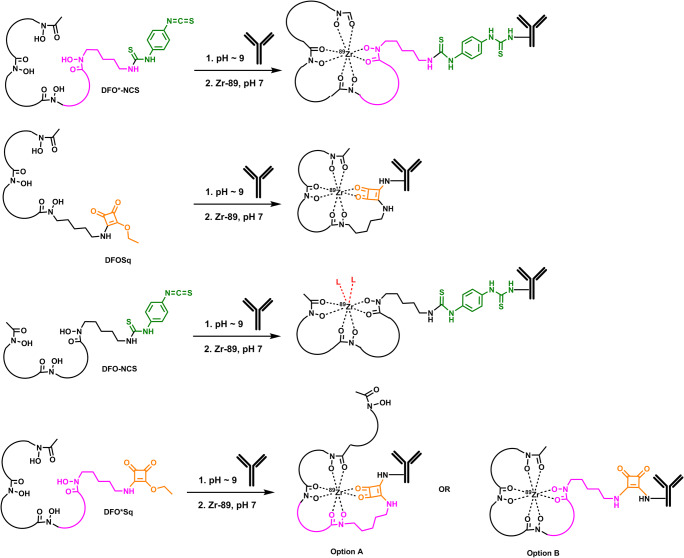
Schematic representation of DFO-NCS, DFO*-NCS, DFOSq and DFO*Sq coupling to a mAb and complexation with ^89^Zr. Option A and B represent two possible molecular representations of ^89^Zr complexation by DFO*Sq

As DFO* exhibits promising preliminary performance, we compare herein the in vitro and in vivo properties of [^89^Zr]Zr-DFO*-mAb conjugates to their [^89^Zr]Zr-DFO analogues using the isothiocyanate and squaramide linker forms. For this purpose, DFO*Sq, a derivative of both DFO* as well as DFOSq, was synthesised and also included in this head-to-head comparison, as this bifunctional chelator might provide additional insight into the mutual contribution of an extra hydroxamate group or squaramide group to ^89^Zr complexation (Fig. 1). To allow comparative in vitro and in vivo studies, [^89^Zr]Zr-DFO*-NCS-trastuzumab, [^89^Zr]Zr-DFOSq-trastuzumab, [^89^Zr]Zr-DFO-NCS-trastuzumab and [^89^Zr]Zr-DFO*Sq-trastuzumab were synthesised based on previously described procedures [23]. Their stability was assessed at 37 °C in serum and formulation solution (± competing chelators such as EDTA, DFO and DFO*). Next, the biodistribution of the four [^89^Zr]Zr-trastuzumab conjugates was assessed in HER2-expressing NCI-N87 tumour-bearing nu/nu mice. Superior performance of DFO* over DFO was further confirmed in a second model using a fast growing, highly internalizing tumour model (EGFR-expressing A431 xenograft). Furthermore, chelator stability ([^89^Zr]Zr-DFO* and [^89^Zr]Zr-DFO) for competing metals was evaluated in vitro using a panel of nine metals either known for their chelating capacity with DFO or their natural abundance in the human body. Finally, to evaluate the practical advantages of stable ^89^Zr coupling for the accurate detection of bone metastases, a mouse model of intratibial breast bone metastases was developed and evaluated using trastuzumab and non-binding control mAb B12 comparing the chelators DFO* and DFO.

## Materials and methods

### General materials

Starting reagents and solvents were obtained from Sigma-Aldrich (DMSO, Na_2_CO_3_, oxalic acid), Merck-Millipore (sucrose, Tween 20®-pharmaceutical grade) or Invitrogen (1 M HEPES). ^89^Zr in 1 mol/L oxalic acid was obtained from Perkin-Elmer (Boston, USA). Water was distilled and deionised using a MilliQ water filtration system (Millipore, USA**).** *p*-Isothiocyanatobenzyl desferrioxamine (*p*-SCN-Bn-deferoxamine (B-705), abbreviated in this publication as DFO-NCS) was purchased from Macrocyclics Inc. (Dallas, TX, USA). DFO*-NCS was synthesised by Mercachem B.V. (Nijmegen, The Netherlands). DFOSq was kindly provided by Prof. Donnelly and Dr. Rudd from the University of Melbourne (Australia) [24], and DFO*Sq was synthesised in house at Genentech Inc. (South San Francisco, CA) [25]. EDTA disodium salt was purchased from Sigma-Aldrich (Saint Louis, USA), DFO-mesylate from Novartis (East Hanover, NJ), and DFO* was either synthesised in house [23] or obtained from ABX (Radeberg, Germany). Human NCI-N87, A431 and SKOV-3 cells lines were obtained from the American Type Culture Collection (ATCC). Trastuzumab (HERCEPTIN®, 21 mg/mL, Roche) and cetuximab (ERBITUX®, 5 mg/mL, Merck) were commercially obtained. The isotype-matched control anti-HIV antibody B12 (53.4 mg/mL) directed against the gp120 of CD4 was graciously provided by the company Lundbeck (Valby, Denmark).

### Synthesis of ^89^Zr-labelled compounds

Synthesis of [^89^Zr]Zr-trastuzumab conjugates, [^89^Zr]Zr-cetuximab conjugates, [^89^Zr]Zr-B12 control conjugates, [^89^Zr]Zr-DFO*, [^89^Zr]Zr-DFO, [^89^Zr]Zr-oxalate, [^89^Zr]Zr-citrate and [^89^Zr]Zr-chloride can be found in the electronic supplementary information.

### Quality controls

Radiochemical purity, protein integrity, binding assays as well as determination of chelator-to-mAb ratio can be found in the electronic supplementary information*.*

### In vitro stability tests

#### Serum stability of radioimmunoconjugates

[^89^Zr]Zr-DFO*-NCS-trastuzumab, [^89^Zr]Zr-DFOSq-trastuzumab, [^89^Zr]Zr-DFO-NCS-trastuzumab and [^89^Zr]Zr-DFO*Sq-trastuzumab (0.2 mg mAb/mL, 10 MBq/mL) were incubated in the presence of human serum (Sigma-Aldrich) following conditions described previously [23]. Five hundred microlitres of each of the radioimmunoconjugates in formulation buffer adjusted to pH 7 with 2 M Na_2_CO_3_ were incubated in triplicate with 500 μL of serum. All samples were incubated over a week at 37 °C in a CO_2_ incubator in the presence of 0.02% NaN_3_ (8 μL, 25 mg/mL in MilliQ water) to maintain sterility. The initial pH was 7, and this stayed constant during the course of the incubation period (measured values between 7.0 and 7.3). Samples were taken before incubation (day 0), and at 1, 3 and 7 days in a laminar flow hood to avoid contamination. Radiochemical purity was determined by SE-HPLC and spin filter analysis (as described in the electronic supplementary information). Immunoreactivity was checked at day 0 and 7 days by a binding assay as described in the electronic supplementary information.

#### EDTA, DFO and DFO* challenge of the radioimmunoconjugates

The four radioimmunoconjugates were challenged with EDTA, DFO and DFO* as described in the electronic supplementary information*.*

#### Metals and other cation challenge of [^89^Zr]Zr-DFO* or [^89^Zr]Zr-DFO

This experiment was adapted from described methods [26, 27] and can be found in the electronic supplementary information*.*

### In vivo experiments

Animal experiments were performed according to the NIH Principles of Laboratory Animal Care, the European Community Council Directive (2010/63/EU) for laboratory animal care and the Dutch Law on animal experimentation (“Wet op de dierproeven,” Stb 1985, 336). The experimental protocol was validated and approved by the central committee for animal experimentation (CCD) and the local committee on animal experimentation of the Amsterdam UMC, Vrije Universiteit Amsterdam. All mice were female nu/nu mice (received at 8 weeks old from either Envigo, The Netherlands or Charles River, USA) and were left for at least 1 week of acclimation before starting any experiment.

#### Biodistribution of [^89^Zr]Zr-trastuzumab conjugates

Biodistribution of [^89^Zr]Zr-DFO*-NCS-trastuzumab, [^89^Zr]Zr-DFOSq-trastuzumab, [^89^Zr]Zr-DFO-NCS-trastuzumab and [^89^Zr]Zr-DFO*Sq-trastuzumab was evaluated in tumour-bearing mice. Forty female nu/nu mice were injected subcutaneously (s.c.) in both flanks with 2 × 10^6^ NCI-N87 cells. Tumour growth was monitored on a daily basis, and tumour volume was assessed with a calliper ((length × width × depth) / 2) at least twice a week as soon as tumours became detectable. When tumours reached an average volume of 100–200 mm^3^, mice were randomised and divided in 8 groups of 5 mice for injection of 100 μg of radioimmunoconjugate in 100–200 μL. Of radioimmunoconjugate, 1.1 MBq was administered intravenously (i.v.) via the retro orbital plexus to animals under anaesthesia with inhalation of 2–4% isoflurane/O_2_. At 2, 24, 48, 72 and 144 h post-injection (p.i.), blood samples were drawn, and at 72 and 144 h p.i., 5 mice per group were anaesthetised, bled, euthanised and dissected. Additionally, among the mice sacrificed at 144 h p.i., two mice per group were imaged at 24, 72 and 144 h p.i. For all mice, blood, tumours and organs of interest were collected, weighed and the amount of radioactivity in each sample was measured in the gamma counter. Radioactivity uptake was calculated as the percentage of the injected dose per gram of tissue (%ID/g).

#### Biodistribution of [^89^Zr]Zr-cetuximab conjugates

Biodistribution of [^89^Zr]Zr-DFO*-NCS-cetuximab and [^89^Zr]Zr-DFO-NCS-cetuximab was evaluated in A431 nu/nu tumour-bearing mice as described in the electronic supplementary information*.*

#### Biodistribution studies in a BT-474 bone metastasis model

To evaluate the performance of DFO* and DFO mAb conjugates in the accurate detection of bone metastases, an intratibial bone metastasis model was developed following a procedure adapted from Campbell et al. [28]. For this purpose, BT-474, a highly proliferating HER2 positive cell line that can present osteoblastic and osteoclastic bone remodelling properties, was employed [29]. One day before surgery until 2 days after surgery, the drinking water of nu/nu mice (11 weeks old) was replaced with water containing carprofen (Rimadyl®) at a concentration of 0.067 mg/mL. On the day of surgery, all mice received 0.1 mg/kg of buprenorphine (Temgesic®) s.c., 30 min before the procedure. Mice were closely monitored during anaesthesia, and an incision was made in the skin to expose the left tibia of each mouse followed by injection of 1.5 × 10^6^ (in 10 μL PBS) luciferase transfected BT-474 cells directly in the tibia. After suture, the same procedure was performed with PBS in the right tibia of every mouse as a negative control. Disease progression was followed by bioluminescence (In Vivo Xtreme, Bruker, The Netherlands) and CT imaging using a preclinical NanoPET/CT (Mediso, Hungary) until tracer injection approximately 6 weeks later. [^89^Zr]Zr-DFO*-NCS-trastuzumab and [^89^Zr]Zr-DFO-NCS-trastuzumab as well as non-binding [^89^Zr]Zr-DFO*-NCS-B12 and [^89^Zr]Zr-DFO-NCS-B12 were administered in a volume of 100–200 μL at a dose of 100 μg containing 2–3 MBq i.v. (*n* = 6 mice per group). PET imaging (*n* = 4/group) was performed at 24, 72 and 144 h p.i. After the last scan, mice were sacrificed followed by assessment of ex vivo biodistribution. To assess “free ^89^Zr” uptake in tumour and non-tumour involved bones, either [^89^Zr]Zr-oxalate (1 MBq), [^89^Zr]Zr-citrate (0.5 MBq) or [^89^Zr]Zr-chloride (0.5 MBq) was injected in 10 other nu/nu mice (*n* = 3–4/group) followed by PET imaging and assessment of ex vivo biodistribution 24 h p.i.

#### Biodistribution of [^89^Zr]Zr-DFO* and [^89^Zr]Zr-DFO

The biodistribution of [^89^Zr]Zr-DFO* and [^89^Zr]Zr-DFO was evaluated in healthy nu/nu mice, 15 min and 1 h after retro-orbital injection of 1–3 μg per mouse, 0.5 MBq, 3 mice per radiolabelled chelator per time point.

### PET imaging

PET imaging was performed with a dedicated small animal NanoPET/CT scanner (Mediso Ltd., Hungary). Mice were anaesthetised by inhalation of 2–4% isoflurane/O_2_ during the whole scanning period (1-h duration per time point). A 5-min CT scan was acquired prior to each PET scan and used for attenuation and scatter correction purposes. Reconstruction was performed using a 3-dimensional reconstruction algorithm (Tera-Tomo; Mediso Ltd.) with four iterations and six subsets, resulting in an isotropic 0.4-mm voxel dimension.

### Statistics

The Grubbs outlier test was used to check and remove outliers, and statistical analysis was performed on the tissue uptake values of the different groups of mice with the Welch’s *t* test. For biodistribution data, the Grubbs test is useful to determine if one value within a group of mice deviates too much (lower or higher) from the mean. Welch *t* test is a *t* test for small groups which does not assume that the variances are equal between the groups. Both assume normal Gaussian distribution of the values. Two-sided significance levels were calculated, and *p* < 0.05 was considered to be statistically significant. All graphs were generated using GraphPad Prism 5.02 software.

## Results

### Synthesis of ^89^Zr-labelled compounds

All radioimmunoconjugates were obtained with a non-decay corrected radiochemical yield between 76 and 85%, a radiochemical purity above 98% and a preserved immunoreactive fraction (> 95%). The chelator to mAb ratio was on average 1 (0.7–1.2) for all radioimmunoconjugates.

### In vitro stability results

#### Serum stability of radioimmunoconjugates

**Fig. 2:**
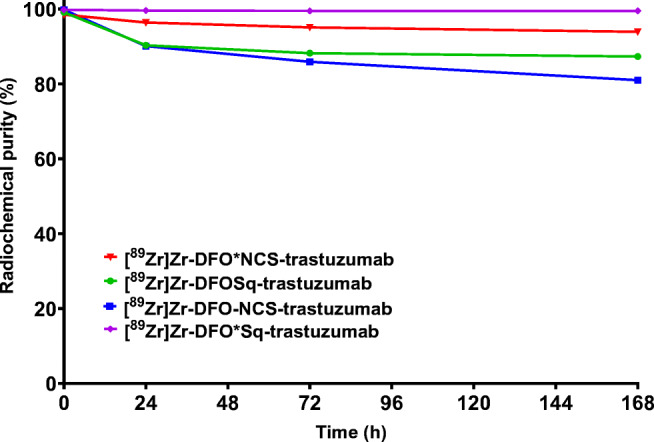
Stability of [^89^Zr]Zr-DFO*-NCS-trastuzumab, [^89^Zr]Zr-DFOSq-trastuzumab, [^89^Zr]Zr-DFO-NCS-trastuzumab, and [^89^Zr]Zr-DFO*Sq-trastuzumab over a week in the presence of serum in a CO_2_ incubator at 37 °C

When the four radioimmunoconjugates were incubated with serum for a week in a CO_2_ incubator at 37 °C, [^89^Zr]Zr-DFO*-NCS-trastuzumab (94 ± 0%) and [^89^Zr]Zr-DFO*Sq-trastuzumab (100 ± 0%) presented a higher radiochemical purity than [^89^Zr]Zr-DFOSq-trastuzumab (87 ± 1%) and [^89^Zr]Zr-DFO-NCS-trastuzumab (81 ± 1%) (Fig. 2). A similar stability trend was exhibited by the immunoreactivity binding assay results: while initially all radioimmunoconjugates presented at least 95% binding, this decreased after 1 week at 37 °C to 90 ± 1 and 96 ± 1% for [^89^Zr]Zr-DFO*-NCS-trastuzumab and [^89^Zr]Zr-DFO*Sq-trastuzumab, respectively, while [^89^Zr]Zr-DFOSq-trastuzumab and [^89^Zr]Zr-DFO-NCS-trastuzumab presented 80 ± 1 and 74 ± 1% immunoreactivity, respectively.

#### EDTA, DFO and DFO* challenge of the radioimmunoconjugates

**Fig. 3:**
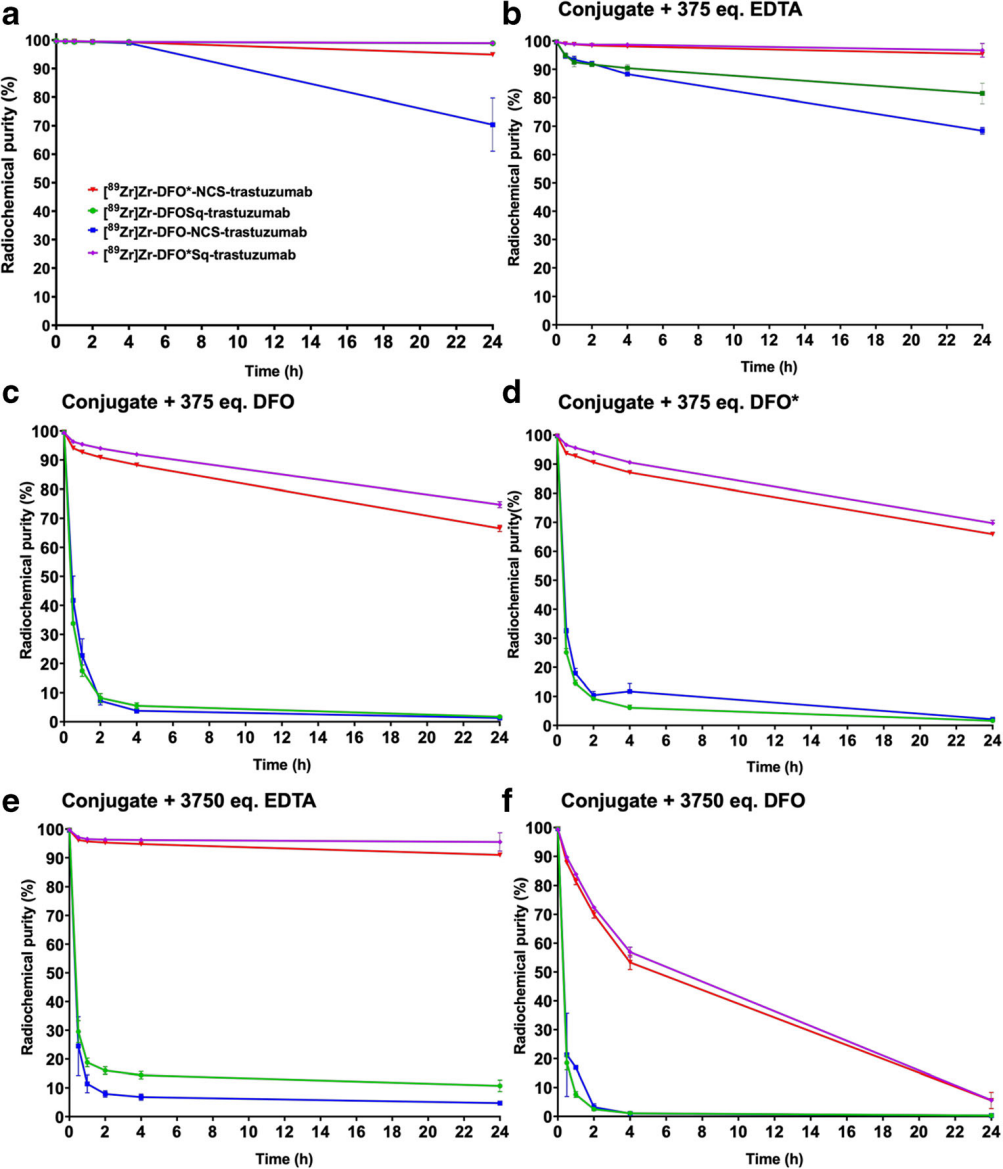
Stability of [^89^Zr]Zr-DFO*-NCS-trastuzumab, [^89^Zr]Zr-DFOSq-trastuzumab, [^89^Zr]Zr-DFO-NCS-trastuzumab and [^89^Zr]Zr-DFO*Sq-trastuzumab incubated for 24 h at 37 °C, at pH 7 (**a**) and when challenged with either 375 equivalents of EDTA (**b**), DFO (**c**) and DFO* (**d**) or 3750 equivalents of EDTA (**e**) and DFO (**f**)

When [^89^Zr]Zr-DFO*-NCS-trastuzumab, [^89^Zr]Zr-DFOSq-trastuzumab, [^89^Zr]Zr-DFO-NCS-trastuzumab and [^89^Zr]Zr-DFO*Sq-trastuzumab were incubated for 24 h at 37 °C in formulation buffer adjusted to pH 7, all four radioimmunoconjugates presented a radiochemical purity above 95% except for [^89^Zr]Zr-DFO-NCS-trastuzumab, which only had a radiochemical purity of 70 ± 9% (Fig. 3a). When challenged with 375 equivalents of EDTA disodium salt, DFO-mesylate or DFO* (Fig. 3b–d), [^89^Zr]Zr-DFO*-NCS-trastuzumab and [^89^Zr]Zr-DFO*Sq-trastuzumab presented a higher radiochemical purity than [^89^Zr]Zr-DFOSq-trastuzumab and [^89^Zr]Zr-DFO-NCS-trastuzumab. With 375 equivalents of EDTA disodium salt, the radioimmunoconjugates comprising DFO* were more than 90% intact (Fig. 3b) while for the DFO radioimmunoconjugates, stability was lower with 82 ± 4% for the DFOSq radioimmunoconjugate followed by 68 ± 1% for the DFO-NCS radioimmunoconjugate. With 375 equivalents DFO-mesylate instead of EDTA disodium salt as challenging chelator, more than 70% intact tracer was left in the case of the DFO* radioimmunoconjugates after incubation for 24 h, while the DFO radioimmunoconjugates were less than 10% intact already after 4 h of incubation (Fig. 3c). With 375 equivalents of DFO*, the effect on stability was very similar to that observed for DFO (Fig. 3d). When the radioimmunoconjugates were challenged with 3750 equivalents of EDTA disodium salt (Fig. 3e) or DFO-mesylate (Fig. 3f), the same trends were observed, although with a more drastic decrease in radiochemical purities, while radiochemical purities of the DFO* radioimmunoconjugates were always higher than those of the comparable DFO radioimmunoconjugates.

Comparable experiments were also performed in formulation buffer at pH 5.5 instead of pH 7.0 (see Fig. S1): [^89^Zr]Zr-DFO*-NCS-trastuzumab and [^89^Zr]Zr-DFO*Sq-trastuzumab presented overall a higher stability than [^89^Zr]Zr-DFOSq-trastuzumab and [^89^Zr]Zr-DFO-NCS-trastuzumab, while in general, all radioimmunoconjugates were less stable at pH 5.5 than at pH 7.0.

#### Metals and other cation challenge of ^89^Zr-DFO* and ^89^Zr-DFO

Finally, the stability of both [^89^Zr]Zr-DFO* and [^89^Zr]Zr-DFO was investigated in challenge experiments with potentially competing metals. Excellent stabilities of > 97% after 168-h incubation were observed when challenged with 10 equivalents of different metals (CoCl_2_, ZnCl_2_, CuCl_2_, MgCl_2_, GaCl_3_, GdCl_3_, AlCl_3_), except when iron (FeCl_3_) and niobium (NbCl_3_) solutions were used (see Table 1). Niobium caused a drastic decrease for both DFO* and DFO with radiochemical purities below 10% within the first hour. Iron induced a gradual decrease in radiochemical purity with 93 ± 1% for [^89^Zr]Zr-DFO* and 89 ± 2% for [^89^Zr]Zr-DFO (significant difference, *p* < 0.05) after 1 week.

**Table 1:** [^89^Zr]Zr-DFO* and [^89^Zr]Zr-DFO stability assessed by iTLC when challenged over a week with 10 equivalents of FeCl_3_ or NbCl_3_, at 37 °C

Radiochemical purity (%)				
	FeCl_3_		NbCl_3_	
Time (h)	^89^Zr-DFO*	^89^Zr-DFO	^89^Zr-DFO	^89^Zr-DFO
0	99.9 ± 0.1	99.6 ± 0.6	99.9 ± 0.1	97.5 ± 0.9
1	95.7 ± 0.3	96.1 ± 0.5	6.6 ± 3.7	9.6 ± 6.2
3	94.6 ± 0.5	92.5 ± 2.8	6.0 ± 1.2	7.7 ± 2.7
6	94.9 ± 1.4	89.7 ± 3.4	6.8 ± 1.2	7.4 ± 1.2
24	93.4 ± 0.5	88.0 ± 2.8	5.7 ± 1.6	5.9 ± 1.5
48	93.9 ± 0.4	87.3 ± 2.4	4.0 ± 2.7	7.3 ± 1.8
72	93.5 ± 0.4	88.6 ± 4.2	6.8 ± 1.8	4.6 ± 1.2
96	93.7 ± 0.8	88.8 ± 3.4	6.7 ± 4.5	11.8 ± 13.8
168	93.0 ± 0.9	88.6 ± 1.7	6.4 ± 1.8	4.3 ± 2.0

### In vivo results

#### Biodistribution of [^89^Zr]Zr-trastuzumab conjugates

**Fig. 4:**
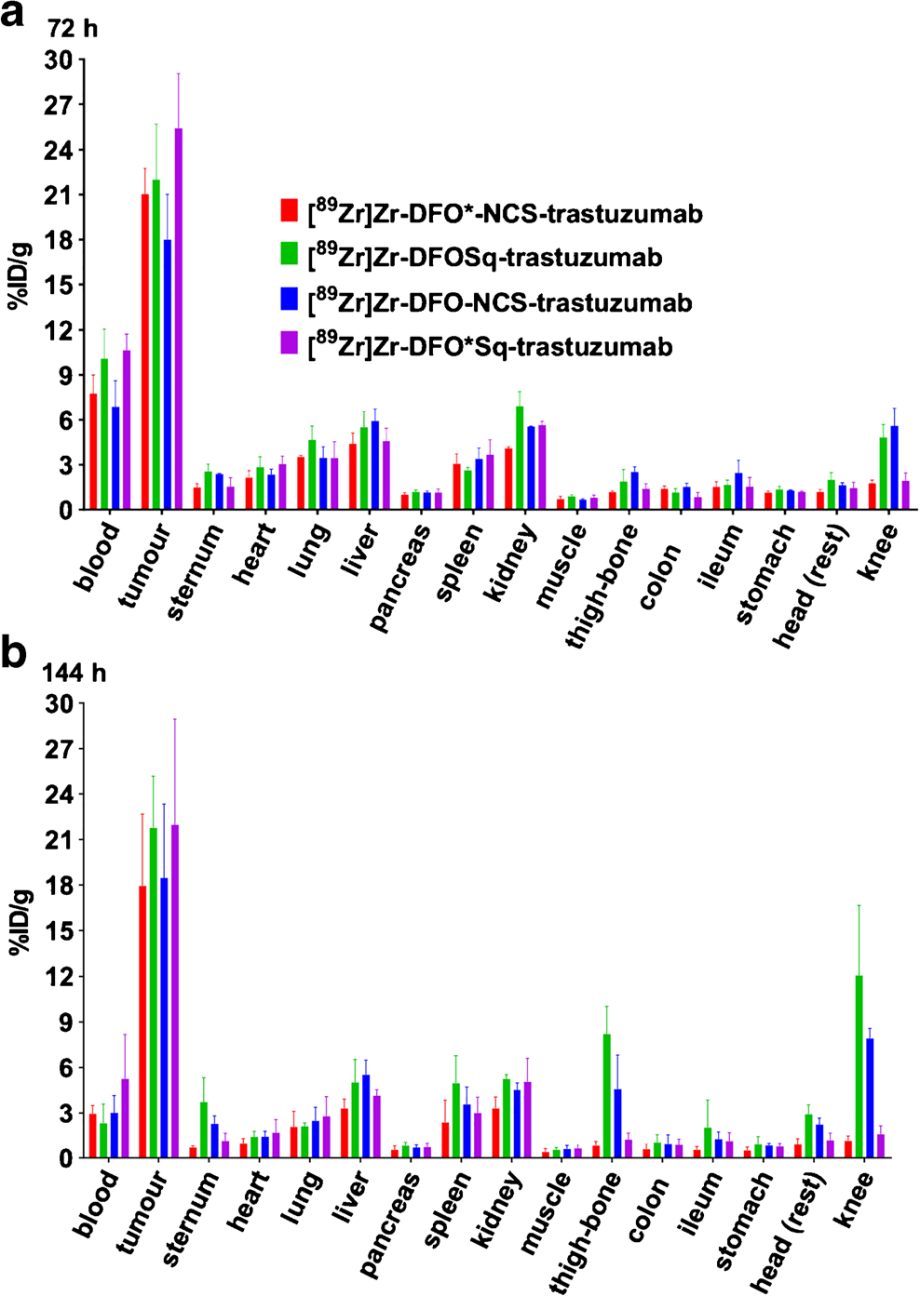
Biodistribution of [^89^Zr]Zr-DFO*NCS-trastuzumab, [^89^Zr]Zr-DFOSq-trastuzumab, [^89^Zr]Zr-DFO-NCS-trastuzumab and [^89^Zr]Zr-DFO*Sq-trastuzumab in N87 tumour-bearing nude mice at 72 (**a**) and 144 h (**b**) p.i. of 100 μg of the respective conjugates. Uptake expressed as %ID/g (mean ± SD, *n* = 5 animals per group)

NCI-N87 tumour sizes were 136.6 ± 64.3 mm^3^ 1 day before tracer injection. In the groups sacrificed at 72 h p.i., the average tumour size was 175.0 ± 103.3 mm^3^ and for the groups sacrificed at 144 h p.i., 238.8 ± 121.9 mm^3^. The biodistribution of the four radioimmunoconjugates at 72 and 144 h p.i. is presented in Fig. 4 and Tables S1 and S2. At 72 h p.i, tumour uptake levels of [^89^Zr]Zr-DFO*-NCS-trastuzumab (21.0 ± 1.7%ID/g), [^89^Zr]Zr-DFOSq-trastuzumab (22.0 ± 3.7%ID/g) and [^89^Zr]Zr-DFO-NCS-trastuzumab (18.0 ± 3.0%ID/g) were similar, while uptake of [^89^Zr]Zr-DFO*Sq-trastuzumab (25.4 ± 3.6%ID/g) was slightly but significantly higher (*p < 0.05*). Of note is that the two squaramide derivatives have a higher retention in the blood with uptake levels of 10.6 ± 1.1 and 10.1 ± 2.0%ID/g for the DFO*Sq and DFOSq tracers, respectively, while the DFO*-NCS and DFO-NCS radioimmunoconjugates presented 7.8 ± 1.2 and 6.9 ± 1.7%ID/g. As a result, the tumour-to-blood ratios were not significantly different for the 4 radioimmunoconjugates. At 144 h p.i., there was no significant difference between [^89^Zr]Zr-DFO*-NCS-trastuzumab, [^89^Zr]Zr-DFOSq-trastuzumab, [^89^Zr]Zr-DFO-NCS-trastuzumab and [^89^Zr]Zr-DFO*Sq-trastuzumab in tumour uptake (17.9 ± 4.7, 21.8 ± 3.5, 18.5 ± 4.9 and 22.0 ± 7.0%ID/g, respectively) and blood levels (3.0 ± 0.6, 2.3 ± 1.3, 3.0 ± 1.1 and 5.3 ± 2.9%ID/g, respectively).

**Fig. 5:**
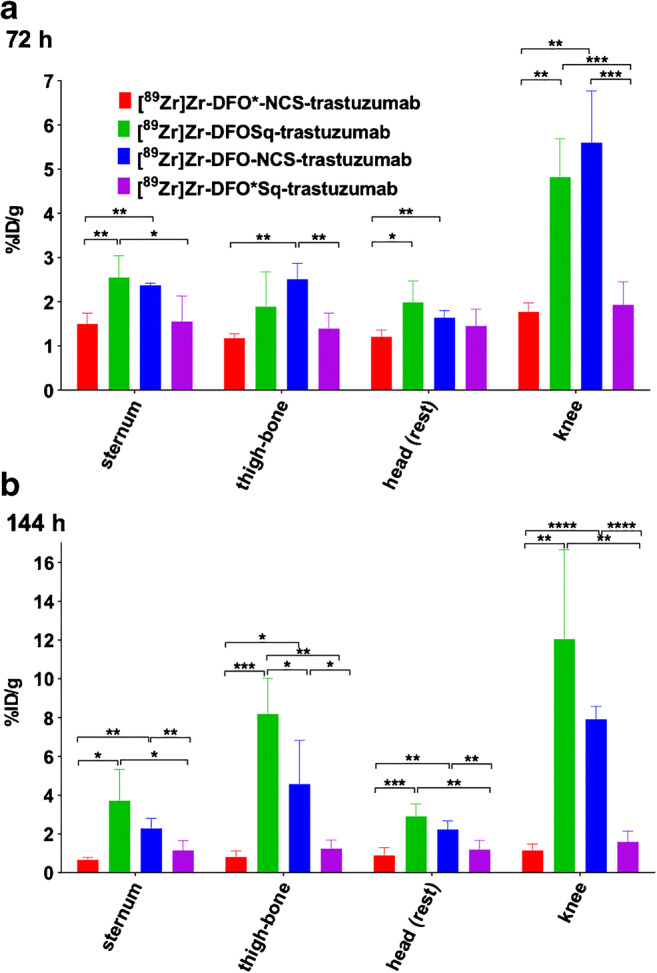
Biodistribution of [^89^Zr]Zr-DFO*NCS-trastuzumab, [^89^Zr]Zr-DFOSq-trastuzumab, [^89^Zr]Zr-DFO-NCS-trastuzumab and [^89^Zr]Zr-DFO*Sq-trastuzumab in N87 tumour-bearing nude mice at 72 (**a**) and 144 h (**b**) p.i. of 100 μg of the respective conjugates. Uptake expressed as %ID/g (mean ± SD, *n* = 5 animals per group). Significant differences between the four constructs are marked with asterisks (*p < 0.05; **p < 0.01; ***p < 0.001; ****p < 0.0001)

**Fig. 6:**
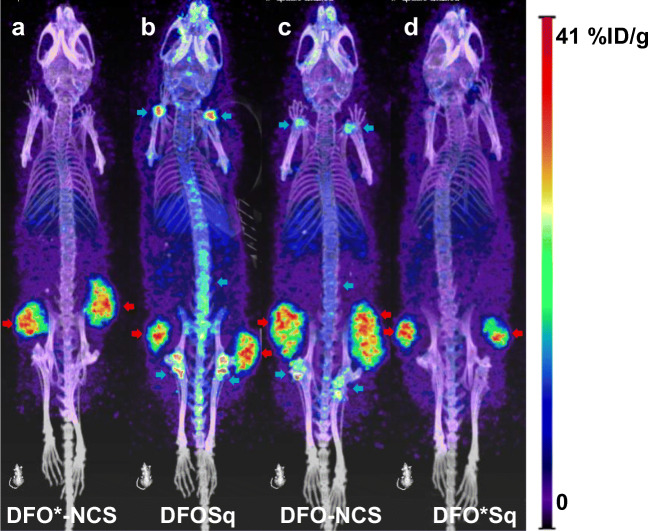
PET images of N87 tumour-bearing mice injected with 110 μg of either [^89^Zr]Zr-DFO*-NCS-trastuzumab (**a**), [^89^Zr]Zr-DFOSq-trastuzumab (**b**), [^89^Zr]Zr-DFO-NCS-trastuzumab (**c**) or [^89^Zr]Zr-DFO*Sq-trastuzumab (**d**) and scanned 144 h p.i. Images are presented as maximum intensity projections (MIPs). Tumours are indicated with red arrows and bone uptake with blue arrows

In the bones (sternum, thigh bone and knee), the two DFO* radioimmunoconjugates presented significantly lower uptake than the two DFO radioimmunoconjugates (Fig. 5). In the knee, the difference was the largest and increased over time in favour of the DFO* radioimmunoconjugates compared with the two DFO radioimmunoconjugates. At 144 h p.i., [^89^Zr]Zr-DFO*-NCS-trastuzumab presented the lowest uptake in the knee (1.2 ± 0.3%ID/g), followed by [^89^Zr]Zr-DFO*Sq-trastuzumab (1.6 ± 0.6%ID/g), [^89^Zr]Zr-DFO-NCS-trastuzumab (7.9 ± 0.7%ID/g) and finally [^89^Zr]Zr-DFOSq-trastuzumab (12.1 ± 4.6%ID/g) (Fig. 5b). PET images were collected at 24, 72 and 144 h p.i., and the 72 and 144-h images were coherent with biodistribution results. Representative PET images obtained 24 and 72 h p.i. of the four radioimmunoconjugates are presented in Fig. S2, and the images obtained at 144 h p.i. are presented in Fig. 6. Notably, the spinal cord, which was not collected for ex vivo tissue distribution, showed higher uptake of the DFO conjugates, especially the DFOSq radioimmunoconjugate.

Concerning metabolic organs, at 72 and 144 h p.i., a tendency was observed for a lower liver uptake of the DFO*-NCS and DFO*Sq radioimmunoconjugates compared with the DFO-Sq and DFO-NCS radioimmunoconjugates, which was only significant at 144 h when the two DFO* radioimmunoconjugates were compared with [^89^Zr]Zr-DFO-NCS-trastuzumab. At 72 and 144 h p.i., spleen uptake was similar for the four radioimmunoconjugates with only a significantly (*p < 0.05*) lower uptake of [^89^Zr]Zr-DFO*NCS-trastuzumab (2.4 ± 1.5%ID/g) compared with [^89^Zr]Zr-DFOSq-trastuzumab (5.0 ± 1.8%ID/g) at 144 h p.i. The uptake of [^89^Zr]Zr-DFO*-NCS-trastuzumab was significantly lower in kidneys, compared with the other three radioimmunoconjugates at 72 h. At 144 h p.i., the difference was still significant in comparison with the DFOSq and DFO-NCS radioimmunoconjugates but not in comparison with [^89^Zr]Zr-DFO*Sq-trastuzumab.

#### Biodistribution of [^89^Zr]Zr-cetuximab conjugates

To further evaluate the comparative performance of DFO* and DFO, the biodistribution of [^89^Zr]Zr-DFO*-NCS-cetuximab and [^89^Zr]Zr-DFO-NCS-cetuximab was compared in A431-bearing mice. Biodistribution was assessed after administration of 100 μg of the radioimmunoconjugates at 24, 72 and 144 h p.i. In Fig. S3, the biodistribution of both radioimmunoconjugates at 72 h p.i. as well as the uptake in bones at 24, 72 and 144 h p.i. is presented. The uptake in all organs at the three time points is presented in Table S3. At 72 and 144 h p.i., a significantly lower uptake in bone was observed for [^89^Zr]Zr-DFO*-NCS-cetuximab compared with [^89^Zr]Zr-DFO-NCS-cetuximab (*p <* 0.05 or *p <* 0.01), which was already present at 24 h p.i., except for the sternum and the head, confirming the superiority of the DFO* form as chelator as observed with trastuzumab.

#### Biodistribution of [^89^Zr]Zr-DFO* and [^89^Zr]Zr-DFO

The biodistribution of [^89^Zr]Zr-DFO* and [^89^Zr]Zr-DFO was assessed in healthy nu/nu mice to determine whether the radiolabelled chelators are directly deposited within bones. By 15 min p.i., almost all activities were excreted in urine (> 660%ID/g), kidneys (23.6 ± 12.1 and 34.7 ± 13.2%ID/g for [^89^Zr]Zr-DFO* and [^89^Zr]Zr-DFO, respectively) and bladder (10.8 ± 6.9 and 10.8 ± 5.5%ID/g, respectively). After 1 h, all other analysed organs contained less than 1% ID/g of both radiolabelled chelators with no significant difference between them (Table S4).

#### Biodistribution studies in an intratibial breast bone metastasis model

**Fig. 7:**
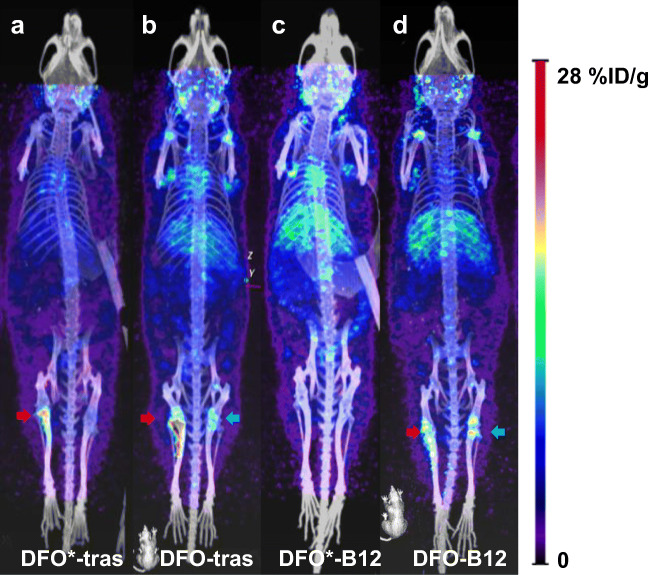
PET images of mice injected with 110 μg of either [^89^Zr]Zr-DFO*-NCS-trastuzumab (**a**), [^89^Zr]Zr-DFO-NCS-trastuzumab (**b**), [^89^Zr]Zr-DFO*-NCS-B12 (**c**) or [^89^Zr]Zr-DFO-NCS-B12 (**d**) and scanned 144 h p.i. All mice had received an intratibial injection of HER2-expressing BT-474 cells in the left leg and PBS in the right leg. Images are presented as maximum intensity projections (MIPs). Uptake in affected tibiae is indicated with red arrows and uptake in contralateral tibiae with blue arrows

**Fig. 8:**
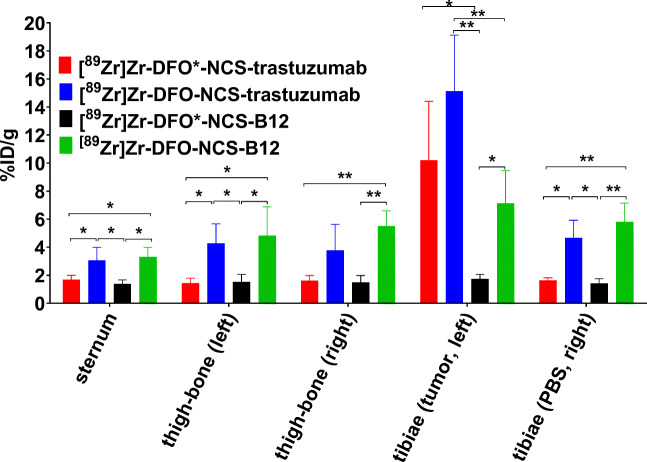
Biodistribution of [^89^Zr]Zr-DFO*-NCS-trastuzumab, [^89^Zr]Zr-DFO-NCS-trastuzumab, [^89^Zr]Zr-DFO*-NCS-B12 and [^89^Zr]Zr-DFO-NCS-B12 in collected bones 144 h p.i. of 100 μg per construct. Uptake expressed as %ID/g (mean ± SD, *n* = 5–6 animals per group). Significant differences between the four constructs are marked with asterisks (*p < 0.05; **p < 0.01)

To determine whether the DFO* chelator provides advantages over DFO in detecting tumours in the bone, a BT-474 bone metastasis model was established and the biodistribution of targeted radioimmunoconjugates ([^89^Zr]Zr-DFO*-NCS-trastuzumab and [^89^Zr]Zr-DFO-NCS-trastuzumab) versus non-targeted radioimmunoconjugates ([^89^Zr]Zr-DFO*-NCS-B12 and [^89^Zr]Zr-DFO-NCS-B12) was performed (Figs. 7 and 8 and Table S5). The uptake in tumour-involved tibiae was significantly lower for [^89^Zr]Zr-DFO*-NCS-B12 (Fig. 7c, left leg) compared with [^89^Zr]Zr-DFO-NCS-B12 (Fig. 7d, left leg): 1.8 ± 0.3%ID/g and 7.1 ± 2.6%ID/g, respectively, at 144 h p.i. In the unaffected tibiae, this was 1.5 ± 0.3%ID/g for [^89^Zr]Zr-DFO*-NCS-B12 (Fig. 7c, right leg), equally low as in affected tibiae (Fig. 7c, left leg), and 5.7 ± 1.4%ID/g for [^89^Zr]Zr-DFO-NCS-B12 (Fig. 7d, right leg). High uptake of ^89^Zr in the tibia in the case of [^89^Zr]Zr-DFO-NCS-B12 radioimmunoconjugates, irrespective of tumour involvement, is likely due to ^89^Zr release, as [^89^Zr]Zr-oxalate, [^89^Zr]Zr-citrate and [^89^Zr]Zr-chloride showed similarly high uptake especially in shoulders, spinal cord and tibiae with non-significant differences between tumour involved and non-involved tibiae (Fig. S4 and Table S6). Mice injected with [^89^Zr]Zr-DFO*-NCS-trastuzumab presented much higher uptake in tumour-involved tibiae (10.2 ± 4.2%ID/g) (Fig. 7a, left leg) than in unaffected tibiae (1.6 ± 0.2%ID/g) (Fig. 7a, right leg). This low uptake of [^89^Zr]Zr-DFO*-NCS-trastuzumab in non-involved tibiae (Fig. 7a, right leg) was similar to the uptake of non-binding [^89^Zr]Zr-DFO*-NCS-B12 in tumour (1.8 ± 0.3%ID/g) (Fig. 7c, left leg) as well as in non-involved tibiae (1.5 ± 0.3%ID/g) (Fig. 7c, right leg). [^89^Zr]Zr-DFO-NCS-trastuzumab showed the highest uptake in tumour-involved tibiae (15.1 ± 4.0%ID/g) (Fig. 7b, left leg); however, the uptake in the unaffected tibiae was also high (4.7 ± 1.3%ID/g) (Fig. 7b, right leg), threefold higher than for the [^89^Zr]Zr-DFO*-NCS-B12 control (Fig. 7c, left and right leg), making DFO less suitable for accurate detection of bone metastases than DFO*.

## Discussion

In this study, we evaluated in depth the in vitro as well as the in vivo performance of DFO* as a future clinical candidate chelator for ^89^Zr-immuno-PET to replace the current gold standard: DFO. The combination of ^89^Zr with DFO leads to unwanted bone uptake in preclinical studies. In clinical studies, this has not been discussed to date, most probably because either uptake in bones is less obvious or because uptake in the case of bone lesions is largely unknown. Furthermore, a new improved chelator has not as yet been directly compared in humans with the current gold standard DFO. The observed preclinical instability has been repeatedly reported in the literature [8, 10] and has led to the development of many new trivalent and tetravalent chelators. In this study, DFO* and DFO radioimmunoconjugates were compared consisting of two different linkers (squaramide and isothiocyanate) between the chelator and mAb. [^89^Zr]Zr-DFO*Sq was developed and included in this comparative study to better understand the coordination chemistry of ^89^Zr chelation in the case of the squaramide linker. In this study, the following experiments have been performed to determine the best second-generation chelator for ^89^Zr: (i) comparison of the in vitro stability of the four DFO* and DFO radioimmunoconjugates in serum at 37 °C as well as in challenge experiments with excess EDTA, DFO or DFO*; (ii) comparison of the four radioimmunoconjugates in vivo in a xenograft model using the mAb trastuzumab; (iii) comparison of the stability of ^89^Zr-chelator complexes when challenged with an excess of metals; (iv) confirmation of DFO* superiority over the current gold standard DFO in a second xenograft model using the mAb cetuximab and finally (v) evaluation of DFO* in comparison with DFO in an intratibial model of tumour bone metastasis. Furthermore, the biodistribution of [^89^Zr]Zr-chelators alone and [^89^Zr]Zr-salts has been determined to evaluate the performance of each chelator, with an emphasis on understanding the mechanism of in vivo ^89^Zr bone uptake.

Our initial goal was to compare DFO*-NCS with DFOSq-based radioimmunoconjugates. In our hands, [^89^Zr]Zr-DFO*-NCS-trastuzumab showed a superior in vitro stability when compared with [^89^Zr]Zr-DFOSq-trastuzumab (Figs. 2 and 3). This was further confirmed in vivo by assessing the uptake in bone; [^89^Zr]Zr-DFO*-NCS-trastuzumab showed a significantly lower uptake than [^89^Zr]Zr-DFOSq-trastuzumab (Figs. 4, 5 and 6). These data are in line with a recent study from Berg et al. [25], in which rhesus monkeys were injected with ^89^Zr-labelled mAbs and followed by total-body PET imaging over a period of 30 days. The results showed that [^89^Zr]Zr-DFOSq-trastuzumab had substantial and significant bone uptake compared with [^89^Zr]Zr-DFO*-NCS-trastuzumab at all imaging time points after day 0 (*p* value *<* 0.05).

In the comparison between DFO*-NCS and DFOSq, DFO-NCS was included as the current clinical gold standard and benchmark for the studies presented herein. While the in vitro and in vivo performance of DFO*NCS appeared clearly superior to DFO-NCS, no obvious advantages were found for DFOSq over DFO-NCS. The superiority of DFO*-NCS in comparison with DFO-NCS became apparent in vitro in serum as well as in chelator challenge experiments. Furthermore, in vivo, a clearly decreased bone uptake using DFO* was observed in two xenograft models with [^89^Zr]Zr-DFO*-trastuzumab and cetuximab. These results are in line with our preliminary results reported previously [23, 30]. Moreover, the impact of more stable ^89^Zr binding was demonstrated in the HER2-positive BT-474 bone model of metastases at 144 h p.i. In tibiae without tumour involvement, the uptake of [^89^Zr]Zr-DFO*-NCS-trastuzumab and the non-binding control [^89^Zr]Zr-DFO*-NCS-B12 was equally low and comparable (1.6 ± 0.2 and 1.5 ± 0.3%ID/g, respectively), which appeared to be similar to the uptake of [^89^Zr]Zr-DFO*-NCS-B12 in tumour-involved tibiae (1.8 ± 0.3%ID/g). Such similar uptake levels as observed with DFO*-NCS might be expected in the case of radioimmunoconjugates that are fully stable in vivo. [^89^Zr]Zr-DFO-NCS-trastuzumab, as well as control [^89^Zr]Zr-DFO-NCS-B12, showed elevated uptake in unaffected tibiae, 4.7 ± 1.3 and 5.7 ± 1.4%ID/g, respectively. This uptake in tibiae was about 3 times higher compared with when DFO*-NCS was used as chelator. Although uptake of the tumour-specific radioimmunoconjugates [^89^Zr]Zr-DFO*-NCS-trastuzumab and [^89^Zr]Zr-DFO-NCS-trastuzumab in tumour-involved tibiae, being 10.2 ± 4.2 and 15.1 ± 4.0%ID/g, respectively, was much higher than in unaffected tibiae, the relatively high ^89^Zr uptake in unaffected tibiae in the case of [^89^Zr]Zr-DFO-NCS-trastuzumab might result in false-positive results in the clinical detection of bone metastases as has to be demonstrated in future studies. Also, in this case, we demonstrated that it is probably not the released ^89^Zr-chelator complex that causes bone uptake as [^89^Zr]Zr-DFO* and [^89^Zr]Zr-DFO were quickly eliminated via the kidneys, but the released ^89^Zr. Although we do not know exactly the complexation status of ^89^Zr after its release from the radioimmunoconjugate in vivo, [^89^Zr]Zr-oxalate, [^89^Zr]Zr-citrate and [^89^Zr]Zr-chloride injected in the BT-474 bone metastasis model in nu/nu mice accumulated in bone, showing high uptake in shoulders, spine and tibiae, the latter irrespective of tumour involvement or not (Fig. S4).

In our study, DFOSq did not perform better than DFO-NCS in vivo*,* while a trend towards higher stability was observed for DFOSq in vitro (Figs. 2 and 3a, b). In chelator challenge experiments using DFO and DFO*, [^89^Zr]Zr-DFOSq-trastuzumab stability was not significantly different from [^89^Zr]Zr-DFO-NCS-trastuzumab; however when EDTA was used, [^89^Zr]Zr-DFOSq-trastuzumab displayed slightly higher stability than [^89^Zr]Zr-DFO-NCS-trastuzumab (Fig. 3b–f). This is in line with the reported in vitro results of Rudd et al. [24]. They observed a higher stability for DFOSq than DFO-NCS in a challenge experiment with 500 equivalents EDTA (pH 7 for 24 h) comparing [^89^Zr]Zr-DFOSqTaur (88% stability) with [^89^Zr]Zr-DFO-p-PhSO_3_H (70%) in water-soluble conditions. In vivo, Rudd et al. [24] showed that the tumour-to-bone ratio was better for DFOSq than for DFO-NCS and also the tumour uptake was higher for DFOSq, but no actual uptake (i.e. in %ID/g) levels in bone were reported.

To evaluate the coordination chemistry of ^89^Zr with DFOSq, we introduced a hybrid chelator, called DFO*Sq, consisting of octadentate chelator DFO* and the same squaramide linker as in DFOSq. While DFOSq and DFO-NCS showed comparable stability in vitro and in vivo, DFO*-NCS and DFO*-Sq exhibited the same superior stability compared with the DFO radioimmunoconjugates, indicating that an extra hydroxamate group is contributing more strongly than a squaramide group to ^89^Zr coordination (Fig. 1, option B). Recently, Holland [31] investigated with density functional theory (DFT) the different coordination isomers for ^89^Zr. While with DFO* eight-coordinate isomers were the most likely, only one of the oxygen atoms of the squaramide moiety of DFOSq seemed to be involved, resulting in a seven-coordinate complex.

In literature, several chelators have been described that aimed at improving coordination of ^89^Zr^4+^, but only few showed promising properties and conjugation to mAbs for in vivo application [7, 32–35]. For example, two bifunctional chelators, *p*-SCN-Bn-HOPO (based on 3,4,3-(LI-1,2-HOPO) [12] and more recently DFO-cyclo*-*p*Phe-NCS [36], have been suggested as second-generation clinical candidate chelators. In the case of *p*-SCN-Bn-HOPO, the synthesis has remained problematic [37], which limits clinical utilisation. DFO-cyclo*-*p*Phe-NCS, reported during the course of our studies, is a racemic compound combining DFO with an additional cyclic hydroxamate moiety and the same linker as used in DFO*-NCS. This chelator demonstrated promising in vitro properties in EDTA and DFO challenge experiments; however, in vivo [^89^Zr]Zr-DFO-cyclo*-NCS-trastuzumab did not show superiority over [^89^Zr]Zr-DFO*-NCS-trastuzumab in HER2+ SKOV-3 tumour-bearing mice. Finally, DOTA, a well-known chelator for other radiometals such as ^111^In, ^177^Lu and ^90^Y, has recently been used to complex ^89^Zr, but has not yet been evaluated as a bifunctional chelator variant [26, 38]. DOTA may be limited by the high temperature (reported temperature 95 °C) required for efficient radiolabelling which may necessitate a prelabeling strategy to generate [^89^Zr]Zr-DOTA-mAb complexes. Furthermore, ^89^Zr in oxalic acid needs to be converted to ^89^Zr in HCl to allow efficient complexation with DOTA.

The question remains as to whether DFO* is the ideal chelator and which linker is most suited for coupling ^89^Zr-chelator to biomolecules. In other words, what are the advantages and disadvantages with respect to (i) solubility of linker-chelator, (ii) reactivity of linker-chelator, (iii) radiolabelling of chelator-mAb complexes, (iv) stability of radioimmunoconjugate and (v) availability of the linker-chelator. Firstly, DFO*-NCS and DFO-NCS are known to be soluble in DMSO but poorly in water, which is also the case for DFO*Sq, while DFOSq is relatively soluble in water (90/10% water/DMSO). Concentrations as used in the conjugation (i.e. 5 mM) are easily achievable in all cases. Thus, solubility is not a limiting factor for conjugation to mAbs in our opinion, since a small percentage of DMSO is allowed in these reactions and coupling is efficient. Also, from a GMP perspective, since DMSO can be easily and efficiently removed from the radioimmunoconjugate before formulation, e.g. during PD-10 purification. Secondly, for modifications of mAbs, DFOSq and DFO*Sq seem to require longer incubation times: overnight at RT, using 3 and 5 equivalents of DFOSq and DFO*Sq, respectively, resulted in 1 DFOSq per mAb molecule on average. This could be improved by increasing the chelator-to-mAb ratio in the conjugation or by increasing the reaction temperature, but to date, no data is available showing to what extent this could improve the conjugation protocol. This constitutes a difference with DFO*-NCS and DFO-NCS for which coupling to mAbs is fast (typically 30 min to 2 h, at 37 °C resulting in case of 3 equivalents of DFO(*)-NCS in one chelator per mAb). Thirdly, radiolabelling of mAbs with ^89^Zr via DFO* or DFO is equally efficient and the same sensitivity is observed for metal impurities: Fe^3+^ and Nb^3+^ can interfere with ^89^Zr binding not only to DFO, but also to DFO*. Although these metals do not affect DFO* radioimmunoconjugate stability in vivo, their presence as impurities might affect the labelling efficiency and the stability of radioimmunoconjugates in vitro, as previously observed by Pandya et al. [26] and Deri et al. [27] with DFO and iron. These findings were not surprising, since DFO is clinically used for the treatment of iron overload, while it has also been used for stable radiolabelling of monoclonal antibodies with ^59^Fe [39] and ^95^Nb, as in studies with [^95^Nb]Nb-DFO-bevacizumab [40, 41]. Fourthly, the in vitro stability of radioimmunoconjugates consisting of DFO* is better than the ones consisting of DFO, and therefore, the need for anti-oxidant additives is less. This is especially important in the case of central tracer manufacturing and distribution to clinical sites. Finally, as DFO*-NCS is now commercially available with a structure very close to the current standard DFO-NCS, its translation to the clinic is facilitated and expected soon.

## Conclusion

In the evaluation of next-generation chelator candidate for clinical ^89^Zr-immuno-PET, DFO* showed a superior in vitro and in vivo performance over the current clinical gold standard DFO, regardless of the linker used (NCS and Sq). [^89^Zr]Zr-DFO*-mAbs appeared the most stable in vivo with the lowest uptake of ^89^Zr in bones, which might be highly relevant to avoid misdiagnosis in the case of bone metastases as was shown herein in an in vivo model of bone metastases. In addition, as DFO*-NCS is now commercially available, the clinical translation is under development.

## Electronic supplementary material

ESM 1 (DOCX 11.1 MB).

## Data Availability

Not applicable.
