# Supplementary material for: Head-to-head comparison of DFO* and DFO chelators: selection of the best candidate for clinical 89Zr-immuno-PET
Source: Eur J Nucl Med Mol Imaging. 2020 Sep 5;48(3):694–707. doi: 10.1007/s00259-020-05002-7 (PMC8036225; doi:10.1007/s00259-020-05002-7)
Supplement: Supplementary file 1 — (DOCX 11.1 MB). [file 259_2020_5002_MOESM1_ESM.docx]

Head to head comparison of DFO* and DFO chelators: selection of the best candidate for clinical ^89^Zr-immuno-PET.

**Running title: Comparison of DFO* and DFO-chelators**

Marion Chomet^1^, Maxime Schreurs^1^, Maria J. Bolijn^1^, Mariska Verlaan^1^, Wissam Beaino ^1^, Kari Brown^1^, Alex J. Poot^1^, Albert D. Windhorst^1^, Herman Gill^2^, Jan Marik^2^, Simon Williams^2^, Joseph Cowell^3^, Gilles Gasser^3^, Thomas L. Mindt^4^, Guus A.M.S van Dongen^1^, Danielle J. Vugts^1,*^.

**Affiliations**

^1^ Amsterdam UMC, Vrije Universiteit Amsterdam, Radiology & Nuclear Medicine, Cancer Center Amsterdam, De Boelelaan 1117, Amsterdam, The Netherlands.

*^2^ Genentech Inc., 1 DNA Way, South San Francisco, California 94080, USA.3*

*^3^Chimie ParisTech, PSL University, CNRS, Institute of Chemistry for Life and Health Sciences, Laboratory for Inorganic Chemical Biology, Paris, France.*

*^4^ Ludwig Boltzmann Institute for Applied Diagnostics, General Hospital Vienna (AKH), Vienna, Austria*

**First Author:** m.chomet@amsterdamumc.nl

**Corresponding Author:** d.vugts@amsterdamumc.nl

**ORCID corresponding author: 0000-0001-7068-8152**

**SUPPLEMENTARY DATA**

**Synthesis of ^89^Zr-labelled compounds**

*[^89^Zr]Zr-trastuzumab conjugates*

[^89^Zr]Zr-DFO*-NCS-trastuzumab and [^89^Zr]Zr-DFO-NCS-trastuzumab were produced as described previously [1] with slight modifications concerning mainly the conjugation time (45 min instead of 30 min) and the formulation buffer (50 mM sodium acetate/200 mM sucrose, pH 5.4-5.6 instead of ﻿20 mM histidine/240 mM sucrose, pH 5.5-5.8) used. Briefly, 5 mg of trastuzumab (0.238 mL, 33 nmol, 21 mg/mL) was diluted to 5 mg/mL with 0.9% NaCl, adjusted to pH 8.9-9.1 with 0.1 M Na_2_CO_3,_ and reacted with 3 equivalents of either DFO*-NCS or DFO-NCS in DMSO (5 mM, 20 µL) at 37 °C for 45 min, as described by Vosjan *et al.* [2]. At the end of incubation, the reaction mixture was applied on a PD-10 column (GE Healthcare Life Sciences) and the product DFO*-NCS-trastuzumab or DFO-NCS-trastuzumab was collected in 2 mL of 50 mM sodium acetate/200 mM sucrose, pH 5.4-5.6 (formulation buffer). The concentration of DFO*-NCS-trastuzumab and DFO-NCS-trastuzumab was determined using trastuzumab as a reference standard with Nanodrop (ThermoFisher^TM^). Radiolabeling of either DFO*-NCS-trastuzumab or DFO-NCS-trastuzumab with ^89^Zr was performed following the same conditions published before [1] but in a 2 mL instead of 1 mL reaction volume. Typically, for *in vivo* studies, 200 μL ^89^Zr (∼60 MBq) in 1 M oxalic acid solution and 90 μL 2M Na_2_CO_3_ were added and reacted for 3 min. Subsequently, the modified mAb (∼1.5 mg) diluted in 0.5 M HEPES buffer (pH 7.0) up to 1.71 mL were added to the reaction mixture and incubated for 60 min at room temperature (RT) under constant agitation. The reaction mixture was then delivered to a PD-10 column and [^89^Zr]Zr-DFO*-NCS-trastuzumab or [^89^Zr]Zr-DFO-NCS-trastuzumab was collected in 2 mL of formulation buffer. Such conditions usually result in a radiolabelling yield of 80 to 85% and a concentration of 0.6 mg/mL checked by HPLC. ﻿Unlabeled mAb is further added for formulation of the reaction mixture to a total mAb dose of 100 μg per mouse.

The modification of trastuzumab with DFOSq was adapted from Rudd *et al.* [3]. Briefly DFOSq was dissolved in 90/10% water/DMSO solution at 2 mg/mL (2.9 mM). 4 mg of trastuzumab (0.191 mL, 26 nmol, 21 mg/mL), 0.182 mL MilliQ water and 0.400 mL of 0.5 M borate buffer (pH 9.0) were mixed, followed by the addition of 3 equivalents of DFOSq (27 µL, 80 nmol) resulting in a total volume of 0.8 mL (5 mg/mL). The reaction was incubated at RT overnight and DFOSq-trastuzumab was purified by centrifugal ultrafiltration (30 kDa, Amicon) for 16 min at 4600 rpm (Rotina 38, Hettich, Germany). The filter was subsequently washed twice, once after addition of 400 µL 0.5 M HEPES (pH 7.0) containing 4% DMSO and finally with 400 µL 0.5 M HEPES (pH 7.0). The concentration of DFOSq-trastuzumab was determined using the trastuzumab reference standard. The radiolabeling, purification and formulation procedures for [^89^Zr]Zr-DFOSq-trastuzumab were the same as for [^89^Zr]Zr-DFO*-NCS-trastuzumab and [^89^Zr]Zr-DFO-NCS-trastuzumab.

For the preparation of [^89^Zr]Zr-DFO*Sq-trastuzumab, modifications to the aforementioned DFOSq conjugation to trastuzumab were applied mainly due to the poor aqueous solubility of DFO*Sq. 5 mg of trastuzumab (0.238 mL, 33 nmol, 21 mg/mL) was mixed with 0.432 mL MilliQ water and 0.500 mL 0.5 M borate buffer (pH 9.0), followed by the addition of 5 equivalents of DFO*Sq (5 mg/mL in DMSO, 29.5 µL, 166.7 nmol) resulting in a total volume of 1.2 mL (4.2 mg/mL). The reaction was incubated at RT overnight and purified as described for DFOSq-trastuzumab. Radiolabeling, purification, and formulation to obtain [^89^Zr]Zr-DFO*Sq-trastuzumab were performed in the same way as for the other radioimmunoconjugates.

*[^89^Zr]Zr-cetuximab conjugates*

[^89^Zr]Zr-DFO*-NCS-cetuximab and [^89^Zr]Zr-DFO-NCS-cetuximab were obtained as described for trastuzumab but with slight modifications. Briefly, 30 mg of cetuximab was buffer exchanged into 0.9% NaCl using PD-10 columns (GE Healthcare Life Sciences, UK). After buffer exchange, cetuximab was concentrated by centrifugal ultrafiltration at 4600 rpm for 16 min (Amicon, 30 kDa). The final concentration was determined using a calibration curve of cetuximab reference standard with Nanodrop. 10 mg of cetuximab (66 nmol) were diluted to 1 mL with 0.9% NaCl, adjusted to pH 8.9-9.1 with 0.1 M Na_2_CO_3,_ and reacted with 3 equivalents of either DFO*-NCS or DFO-NCS in DMSO (5 mM, 40 µL) at 37 °C for 45 min. The reaction mixture was purified on a PD-10 column and the product DFO*-NCS-cetuximab or DFO-NCS-cetuximab was collected in 2 mL formulation buffer. The concentration of modified DFO*-NCS-cetuximab and DFO-NCS-cetuximab was determined against a cetuximab reference standard. Radiolabeling with ^89^Zr was performed for 60 min at RT in a 2 mL reaction at pH 7 using 0.5 M HEPES (pH 7.0). Each reaction mixture was applied to a PD-10 column and [^89^Zr]Zr-DFO*-NCS-cetuximab or [^89^Zr]Zr-DFO-NCS-cetuximab were collected in 2 mL of the formulation buffer.

*[^89^Zr]Zr-B12 control conjugates*

B12 was used in the *in vivo* bone metastases model study as a non-binding mAb to assess non-specific uptake in bones in a mouse model of breast bone metastases. For this purpose, B12 was modified and radiolabelled with either DFO*-NCS or DFO-NCS following the same conditions described for trastuzumab to obtain [^89^Zr]Zr-DFO*-NCS-B12 and [^89^Zr]Zr-DFO-NCS-B12.

*[^89^Zr]Zr-DFO* and [^89^Zr]Zr-DFO*

DFO* was dissolved in DMSO and DFO-mesylate in MilliQ water at a concentration of 5 mM. The chelators were radiolabelled in a total volume of 0.3 mL, starting with approximately 10 MBq of ^89^Zr in 30 μL 1 M oxalic acid, 71 µL 0.9 % NaCl, 13 µL 2 M Na_2_CO_3_, 36 μL of chelator (100 µg, 180 nmol) and 150 µL of 0.5 M HEPES (pH 7.0). After a 30 min incubation at RT, both products were diluted with 0.6-0.7 mL 0.9% NaCl and loaded on a C18-Light Sep-Pak cartridge (Waters, The Netherlands), pre-conditioned with 10 mL methanol followed by 30 mL MilliQ. The Sep-Pak cartridge was subsequently washed with 50 mL 0.9% NaCl followed by 50 mL MilliQ, then dried with 100 mL air. The products were eluted with pure ethanol in 200 µL fractions. Radiochemical purity was determined by HPLC and iTLC, concentrations were assessed by HPLC as described below, and the final products were formulated in 0.9% NaCl to less than 10% ethanol volume prior to retro orbital injection in healthy nu/nu mice (0.5 MBq; 1-3 µg per mouse, 3 mice per radioimmunoconjugate). For analysis of [^89^Zr]Zr-DFO* samples, a C18 Alltima 5 μm column (Grace^TM^) (4.6 × 250 mm) was used, with a gradient starting from 80% water/0.1%TFA and 20% MeCN/0.1% TFA at t=0 to 100% MeCN/0.1% TFA at 20 min with a flow of 1 mL/min. (Rt_DFO*_= 12.7 min). Radiochemical purity was assessed using a radioactivity detector and concentration was assessed at 210 nm. For analysis of [^89^Zr]Zr-DFO samples, an X-Terra 5 μm column (Waters) (3.9 × 150 mm) was employed with a mobile phase consisting of 10 mM sodium phosphate buffer, pH 6 (A) and 100% MeCN (B) and with the following gradient: 5% B from 0-5 min and 5-35% B from 5-21 min (Rt_DFO_ = 9.2 min, flow rate = 1 mL/min). Additionally, radiochemical purity of [^89^Zr]Zr-DFO* and [^89^Zr]Zr-DFO was also assessed using iTLC SG strips (Agilent Technologies, Santa Clara, CA, USA) with 5 mL 50 mM EDTA (pH = 5) as mobile phase (11.5 cm strip; 6 min run time). Two microliters of product were applied to the base of the strip and placed vertically in the eluent. After 6 min, the strip was removed, cut into 11 sections of 1 cm, and all sections put in separate tubes for counting in the gamma counter. After background correction, purity was determined by dividing the counts of the first 9 fractions by the total counts. At the introduction of this method it was first validated that free ^89^Zr migrates to the solvent front (retardation factor Rf = 1) and ^89^Zr-complexes remain at the start (Rf = 0). Both [^89^Zr]Zr-DFO* and [^89^Zr]Zr-DFO were obtained with high radiochemical purity (> 98%), and a non-decay corrected radiochemical yield of 39 ± 9% for [^89^Zr]Zr-DFO* (n=5) and 22 ± 1% for [^89^Zr]Zr-DFO (n=2).

*[^89^Zr]Zr-oxalate, [^89^Zr]Zr-citrate and [^89^Zr]Zr-chloride*

To assess the biodistribution of “free” ^89^Zr in a mouse model of intratibial BT-474 bone metastases, solutions of ^89^Zr^4+^ as [^89^Zr]Zr-oxalate, [^89^Zr]Zr-citrate and [^89^Zr]Zr-chloride in water were prepared. 1-2 µL of ^89^Zr in 1 mol/L oxalic acid were diluted to 200 µL in 0.9% NaCl, adjusted to pH 5.5 with 2 M Na_2_CO_3_ and injected *in vivo* as [^89^Zr]Zr-oxalate. 1-2 µL of ^89^Zr in 1 mol/L oxalic acid were diluted to 200 µL in a 0.02 M citrate buffer, adjusted to pH 5.5 with 2 M Na_2_CO_3_ and injected *in vivo* as [^89^Zr]Zr-citrate. Finally, [^89^Zr]Zr-chloride was prepared following a procedure described by Pandya *et al.* [4] using a Sep-Pak Accell Plus QMA light cartridge (Waters, Germany).

**Quality controls**

*Radiochemical purity and protein integrity*

Radioimmunoconjugates were checked for their radiochemical purity by size-exclusion high performance liquid chromatography (SE-HPLC) and spin filter analysis as described before [1]. In short, a Jasco HPLC system was equipped with a Superdex® 200 Increase 10/300 GL (30 cm × 10 mm, 8.6 μm) size exclusion column (GE Healthcare Life Sciences) and a guard column using 0.05 M phosphate buffer/0.15 M NaCl/0.01 M NaN_3_ (pH 6.7) as mobile phase with a run time of 40 min at 0.75 mL/min. The radioactivity was monitored with an inline NaI(Tl) radioactivity detector (Raytest Sockett). The radioimmunoconjugates eluted at approximately 15 min and ^89^Zr/^89^Zr-chelator at around 27 min. The radiochemical purity was expressed as the percentage of the area under curve of the radiolabelled product compared to the total area on the radioactive channel. Antibody concentration and integrity were assessed on the same SE-HPLC system using the areas under curve on the UV channel at 280 nm. The concentration was determined against a calibration curve of the cold compound.

The radiochemical purity of each radioimmunoconjugate was also assessed by spin filter analysis following a described procedure [1]. A wash buffer was prepared consisting of formulation buffer containing 5% DMSO. Four microliters of product diluted to 100 µL with wash buffer were pipetted onto a 30 kDa cut-off spin filter (Ultracel YM-30, regenerated cellulose, 30 kDa cut-off, Merck Millipore), which was subsequently centrifuged at 14000 rpm for 7 min (Eppendorf 5430). The filter was then washed with 100 µL of the wash buffer, and spun again for 7 min at 14000 rpm, before being washed a second time with 100 µL buffer and spun down again at the same settings. Subsequently, the filter and combined filtrate were counted separately in a gamma counter (LKB Wallac Gamma Counter, model 1282 Compugamma CS), and radiochemical purity was determined by calculating the ratio of counts on the filter (with background subtracted) to the total number of counts (filtrate plus the counts on the filter (with background subtracted)).

*Binding assays*

Immunoreactivity of the radioimmunoconjugates to be used for *in vivo* studies was determined following a procedure described by Lindmo *et al.* [5]. Briefly SKOV-3 (HER2) or A431 (EGFR) cells were serially diluted at concentrations ranging from 0.5 × 10^7^ to 3.13 × 10^5^/mL in phosphate buffered saline (PBS, Fresenius Kabi, Austria) containing 1% of bovine serum albumin (BSA, Sigma-Aldrich). The lowest cell concentration was tested twice, with and without addition of cold mAb to assess non-specific binding (either 2 μL of trastuzumab or 5 μL of cetuximab). The assay was performed in triplicate and 0.5 mL of the radiolabelled product (diluted to a concentration of 8.5 ng/mL for trastuzumab and 10 ng/mL for cetuximab) was added to all tubes. The tubes were placed in a head-over-head rotator at 4 ^o^C overnight and subsequently centrifuged for 5 min at 1500 rpm. Finally, supernatants and pellets were separated and counted in a gamma counter in order to determine the immunoreactive fraction, i.e. the percentage of radioactive mAb bound to the cells.

*Determination of chelator-to-mAb ratio*

The production of chelator-mAb conjugates was developed to achieve a chelator-to-mAb ratio of 1:1. DFO*Sq and DFOSq modified mAb samples were diluted to a concentration of 1 mg/mL in PBS and deglycosylated with PNGase F (Sigma-Aldrich) with 2 units/μg of enzyme for 100 µg of antibody. Samples were incubated at 37 °C for 24 h and analyzed by SE-MS as described by Sijbrandi *et al.* [6]*.* DFO*-NCS and DFO-NCS modified mAb samples were prepared as described above. However, SE-MS was not possible, since the thiourea bond in the conjugates was not stable under the applied analysis conditions. Therefore, to evaluate the chelator-to-mAb ratio, the chelator-mAb conjugate was not purified from unreacted chelator. Instead, a sample was taken for radiolabeling with ^89^Zr (2-3 MBq) as described above. The radiolabeling yield of the purified product (radioactivity in eluent divided by total radioactivity, typical values are 30-35%) was then used to calculate the chelator-to-mAb ratio.

***In vitro* stability tests**

*EDTA, DFO, and DFO* challenge of the radioimmunoconjugates*

The four radioimmunoconjugates were formulated in 50 mM sodium acetate/200 mM sucrose (pH 5.4-5.6) at a concentration of 0.2 mg/mL, 8 MBq/mL. Formulated products (0.67 nmol, 0.5 mL) were challenged with 250 and 2500 nmol (375- and 3750-fold excess compared to the mAb conjugates with an average chelator-to-mAb ratio of 1) of either EDTA disodium salt or DFO-mesylate (250 µL of 1 mM or 10 mM respective chelator solution in MilliQ water) or 250 nmol (375-fold excess) of DFO* (250 µL, 10 mM in DMSO further diluted to 1 mM in MilliQ water) in a total volume of 750 µL at pH 5.5 and 7. As a control, all four radioimmunoconjugates (0.67 nmol, 0.5 mL) were also incubated in the absence of chelator (total volume 0.5 mL). All samples were incubated at 37 °C, at pH 5.5 and 7.0 (pH of the solution adjusted with 2 M Na_2_CO_3_ and remained stable during the experiment). Radiochemical purity was assessed at 0, 0.5, 1, 2, 4 and 24 h incubation by centrifugal ultrafiltration (Amicon, 30 kDa) as described in the quality controls using a wash buffer containing 5% DMSO and 95% 20 mM histidine/240 mM sucrose buffer/0.01% Tween 20. The filtrate contained free ^89^Zr/^89^Zr-chelator, while the radioimmunoconjugate remained on the filter. All challenges were performed in triplicate.

*Metals and other cations challenge of [^89^Zr]Zr-DFO* or [ ^89^Zr]Zr-DFO*

This experiment was adapted from described methods [7,8]. 0.344 µmol DFO* (1.5 mL, obtained from a 10 mM solution in DMSO and further diluted to 229 µM in MilliQ water) or DFO-mesylate (1.5 mL, at a concentration of 229 µM in MilliQ water) were radiolabelled with ^89^Zr (diluted in 1 M oxalic acid (300 µL, 6 MBq)) in the presence of 2 M Na_2_CO_3_ (135 µL) and 0.5 M HEPES (1.5 mL) at pH 7 and RT for 15 min. The solutions were then split into samples of 200 µL containing 0.02 µmol chelator and incubated with a 10-fold excess of competing metals or cation salts (200 µL of 1 mM solution in MilliQ water of either FeCl_3_, CoCl_2_, ZnCl_2_, CuCl_2_, MgCl_2_, GaCl_3_, GdCl_3_, AlCl_3_, NbCl_3_ and in an additional sample NaCl was used as a control). The samples were incubated for a week at 37 °C in a thermomixer while shaking (550 rpm). The pH remained stable over time. Two microliter samples were taken over time (0, 1, 3, 24, 48, 72, 96 and 168 h) and the radiochemical purity was determined by iTLC. The iTLC method was validated after checking the retardation factor of ^89^Zr compared to ^89^Zr-DFO using various buffers: 20 mM citric acid/50 mM EDTA pH 4.9; 20 mM citric acid/50 mM DTPA pH 4.9; 100 mM EDTA pH 4.9; and 50 mM DTPA pH 4.9. A buffer of 50 mM DTPA at pH 4.9 demonstrated the best ability to separate the radioactive species and was thus selected. The experiments were performed in triplicate.

***In vivo* experiments**

*Biodistribution of [^89^Zr]Zr-cetuximab conjugates*

Biodistribution of [^89^Zr]Zr-DFO*-NCS-cetuximab and [^89^Zr]Zr-DFO-NCS-cetuximab was evaluated in A431 nu/nu tumour-bearing mice. Because of the heterogeneous and fast growth of A431 tumours, 4 donor mice were first injected s.c in both flanks with 2.5 × 10^6^ A431 cells. After 2 weeks of tumour growth, the four donor mice were sacrificed, the viable parts of the tumours were cut into small pieces of approximately the same size, kept briefly in cell culture medium before transplantation of one piece of tumour in each flank of 30 new nu/nu mice by a small incision under the skin while being kept under anesthesia. After two weeks, mice were randomized and divided into 6 groups of 5 mice for injection of 100 μg of [^89^Zr]Zr-DFO*-NCS-cetuximab or [^89^Zr]Zr-DFO-NCS-cetuximab in 100-200 µL. 1.1 MBq of radioimmunoconjugate was administered intravenously (i.v.) via the retro orbital plexus to animals under anesthesia (by the inhalation of 2-4% isoflurane/O_2_). At 24, 72 and 144 h p.i. 5 mice per group were anesthetized, bled, euthanized and dissected. Additionally, among the mice sacrificed at 144 h p.i., two mice were imaged at 24, 72 and 144 h p.i. Biodistribution was assessed as described for the trastuzumab conjugates (%ID/g).

**Table S1.** Biodistribution of [^89^Zr]Zr-DFO*NCS-trastuzumab, [^89^Zr]Zr-DFOSq-trastuzumab, [^89^Zr]Zr-DFO-NCS-trastuzumab, and [^89^Zr]Zr-DFO*Sq-trastuzumab in N87 tumour-bearing nude mice, 72 h after administration of 100 µg of conjugate. Results are expressed as mean (%ID/g) ± sd (n=5 mice per group)

| **72h** | **[^89^Zr]Zr-DFO*-NCS- trastuzumab** | | | **[^89^Zr]Zr-DFOSq-trastuzumab** | | | **[^89^Zr]Zr-DFO-NCS-trastuzumab** | | | **[^89^Zr]Zr-DFO*Sq-trastuzumab** | | |
| --- | --- | --- | --- | --- | --- | --- | --- | --- | --- | --- | --- | --- |
| blood | 7.8 | ± | 1.2 | 10.1 | ± | 2.0 | 6.9 | ± | 1.7 | 10.6 | ± | 1.1 |
| urine | 9.8 | ± | 1.1 | 2.7 | ± | 1.4 | 1.5 | ± | 0.5 | 6.6 | ± | 2.4 |
| skin | 3.3 | ± | 0.3 | 4.5 | ± | 0.4 | 4.0 | ± | 0.5 | 3.9 | ± | 0.7 |
| bladder | 4.1 | ± | 0.8 | 3.7 | ± | 0.6 | 4.1 | ± | 0.4 | 4.3 | ± | 0.8 |
| tumour | 21.0 | ± | 1.7 | 22.0 | ± | 3.7 | 18.0 | ± | 3.0 | 25.4 | ± | 3.6 |
| sternum | 1.5 | ± | 0.2 | 2.6 | ± | 0.5 | 2.4 | ± | 0.1 | 1.6 | ± | 0.6 |
| heart | 2.2 | ± | 0.5 | 2.8 | ± | 0.7 | 2.3 | ± | 0.4 | 3.0 | ± | 0.5 |
| lung | 3.5 | ± | 0.1 | 4.7 | ± | 0.9 | 3.5 | ± | 0.8 | 3.5 | ± | 1.1 |
| liver | 4.4 | ± | 0.7 | 5.5 | ± | 1.1 | 5.9 | ± | 0.8 | 4.6 | ± | 0.9 |
| pancreas | 1.0 | ± | 0.1 | 1.2 | ± | 0.1 | 1.2 | ± | 0.1 | 1.2 | ± | 0.2 |
| spleen | 3.1 | ± | 0.7 | 2.6 | ± | 0.2 | 3.4 | ± | 0.7 | 3.7 | ± | 1.0 |
| kidney | 4.1 | ± | 0.1 | 6.9 | ± | 1.0 | 5.6 | ± | 0.0 | 5.7 | ± | 0.3 |
| muscle | 0.7 | ± | 0.2 | 0.9 | ± | 0.1 | 0.7 | ± | 0.1 | 0.8 | ± | 0.2 |
| thigh-bone | 1.2 | ± | 0.1 | 1.9 | ± | 0.8 | 2.5 | ± | 0.4 | 1.4 | ± | 0.4 |
| colon | 1.4 | ± | 0.2 | 1.2 | ± | 0.3 | 1.5 | ± | 0.2 | 0.9 | ± | 0.3 |
| colon content | 2.4 | ± | 0.5 | 0.7 | ± | 0.2 | 1.9 | ± | 0.7 | 0.7 | ± | 0.3 |
| ileum | 1.5 | ± | 0.3 | 1.7 | ± | 0.3 | 2.5 | ± | 0.9 | 1.6 | ± | 0.6 |
| ileum content | 0.8 | ± | 0.1 | 0.6 | ± | 0.4 | 1.0 | ± | 0.5 | 0.6 | ± | 0.4 |
| stomach | 1.2 | ± | 0.1 | 1.4 | ± | 0.2 | 1.3 | ± | 0.0 | 1.2 | ± | 0.1 |
| stomach content | 0.7 | ± | 0.3 | 0.2 | ± | 0.1 | 0.3 | ± | 0.2 | 0.2 | ± | 0.1 |
| brain | 0.2 | ± | 0.0 | 0.3 | ± | 0.1 | 0.2 | ± | 0.1 | 0.3 | ± | 0.1 |
| head | 1.2 | ± | 0.2 | 2.0 | ± | 0.5 | 1.6 | ± | 0.2 | 1.5 | ± | 0.4 |
| knee | 1.8 | ± | 0.2 | 4.8 | ± | 0.9 | 5.6 | ± | 1.2 | 1.9 | ± | 0.5 |

**Table S2.** Biodistribution of [^89^Zr]Zr-DFO*NCS-trastuzumab, [^89^Zr]Zr-DFOSq-trastuzumab, [^89^Zr]Zr-DFO-NCS-trastuzumab and [^89^Zr]Zr-DFO*Sq-trastuzumab in N87 tumour-bearing nude mice, 144 h after administration of 100 µg of conjugate. Results are expressed as mean (%ID/g) ± sd (n=5 mice per group)

| **144h** | **[^89^Zr]Zr-DFO*-NCS- trastuzumab** | | | **[^89^Zr]Zr-DFOSq-trastuzumab** | | | **[^89^Zr]Zr-DFO-NCS-trastuzumab** | | | **[^89^Zr]Zr-DFO*Sq-trastuzumab** | | |
| --- | --- | --- | --- | --- | --- | --- | --- | --- | --- | --- | --- | --- |
| blood | 3.0 | ± | 0.6 | 2.3 | ± | 1.3 | 3.0 | ± | 1.1 | 5.3 | ± | 2.9 |
| urine | 4.0 | ± | 1.3 | 2.2 | ± | 0.9 | 1.0 | ± | 0.2 | 2.5 | ± | 1.3 |
| skin | 1.8 | ± | 0.5 | 2.5 | ± | 0.2 | 2.6 | ± | 0.3 | 3.0 | ± | 1.4 |
| bladder | 1.6 | ± | 0.1 | 2.2 | ± | 0.3 | 1.9 | ± | 0.2 | 2.8 | ± | 1.3 |
| tumour | 17.9 | ± | 4.7 | 21.8 | ± | 3.5 | 18.5 | ± | 4.9 | 22.0 | ± | 7.0 |
| sternum | 0.7 | ± | 0.1 | 3.7 | ± | 1.6 | 2.3 | ± | 0.5 | 1.2 | ± | 0.5 |
| heart | 1.0 | ± | 0.3 | 1.4 | ± | 0.4 | 1.4 | ± | 0.4 | 1.7 | ± | 0.9 |
| lung | 2.1 | ± | 1.0 | 2.1 | ± | 0.2 | 2.5 | ± | 0.9 | 2.8 | ± | 1.3 |
| liver | 3.3 | ± | 0.6 | 5.0 | ± | 1.5 | 5.5 | ± | 1.0 | 4.1 | ± | 0.4 |
| pancreas | 0.5 | ± | 0.3 | 0.8 | ± | 0.3 | 0.7 | ± | 0.2 | 0.7 | ± | 0.3 |
| spleen | 2.4 | ± | 1.5 | 5.0 | ± | 1.8 | 3.6 | ± | 1.2 | 3.0 | ± | 1.1 |
| kidney | 3.3 | ± | 0.8 | 5.2 | ± | 0.3 | 4.5 | ± | 0.5 | 5.1 | ± | 1.6 |
| muscle | 0.4 | ± | 0.2 | 0.5 | ± | 0.2 | 0.6 | ± | 0.2 | 0.6 | ± | 0.3 |
| thigh-bone | 0.8 | ± | 0.3 | 8.2 | ± | 1.8 | 4.6 | ± | 2.3 | 1.2 | ± | 0.4 |
| colon | 0.6 | ± | 0.3 | 1.0 | ± | 0.5 | 1.0 | ± | 0.6 | 0.9 | ± | 0.4 |
| colon content | 1.3 | ± | 0.5 | 0.3 | ± | 0.1 | 1.2 | ± | 0.2 | 0.4 | ± | 0.1 |
| ileum | 0.5 | ± | 0.2 | 2.0 | ± | 1.8 | 1.3 | ± | 0.5 | 1.1 | ± | 0.6 |
| ileum content | 0.5 | ± | 0.3 | 0.6 | ± | 0.6 | 0.5 | ± | 0.1 | 0.4 | ± | 0.2 |
| stomach | 0.5 | ± | 0.2 | 0.9 | ± | 0.5 | 0.8 | ± | 0.2 | 0.8 | ± | 0.2 |
| stomach content | 0.1 | ± | 0.1 | 0.1 | ± | 0.0 | 0.2 | ± | 0.2 | 0.1 | ± | 0.0 |
| brain | 0.1 | ± | 0.0 | 0.2 | ± | 0.1 | 0.2 | ± | 0.1 | 0.2 | ± | 0.1 |
| head | 0.9 | ± | 0.4 | 2.9 | ± | 0.6 | 2.2 | ± | 0.4 | 1.2 | ± | 0.5 |
| knee | 1.2 | ± | 0.3 | 12.1 | ± | 4.6 | 7.9 | ± | 0.7 | 1.6 | ± | 0.6 |

**Table S3.** Biodistribution of [^89^Zr]Zr-DFO*-NCS-cetuximab and [^89^Zr]Zr-DFO-NCS-cetuximab in A431 tumour-bearing nude mice, 24, 72 and 144 h after administration of 100 µg of conjugate. Results are expressed as mean (%ID/g) ± sd (n=5 mice per group)

|  | **24 h** | | | | | | **72 h** | | | | | | | | **144 h** | | | | | | |
| --- | --- | --- | --- | --- | --- | --- | --- | --- | --- | --- | --- | --- | --- | --- | --- | --- | --- | --- | --- | --- | --- |
|  | **[^89^Zr]Zr-DFO*-NCS-cetuximab** | | | **[^89^Zr]Zr-DFO-NCS-cetuximab** | | | | **[^89^Zr]Zr-DFO*-NCS-cetuximab** | | | **[^89^Zr]Zr-DFO-NCS-cetuximab** | | | **[^89^Zr]Zr-DFO*-NCS-cetuximab** | | | | **[^89^Zr]Zr-DFO-NCS-cetuximab** | | |  |
| blood | 10.2 | ± | 2.6 | 8.3 | ± | 1.9 | | 5.0 | ± | 1.2 | 4.0 | ± | 1.6 | 0.1 | | ± | 0.0 | 0.3 | ± | 0.1 |  |
| urine | 7.5 | ± | 3.7 | 1.4 | ± | 0.6 | | 6.2 | ± | 2.1 | 0.8 | ± | 0.3 | 1.9 | | ± | 0.6 | 0.5 | ± | 0.1 |  |
| skin | 3.9 | ± | 0.6 | 3.4 | ± | 0.5 | | 2.2 | ± | 0.4 | 2.8 | ± | 0.6 | 0.6 | | ± | 0.1 | 2.5 | ± | 0.9 |  |
| bladder | 3.1 | ± | 0.5 | 2.8 | ± | 0.6 | | 2.0 | ± | 0.3 | 2.0 | ± | 0.5 | 0.4 | | ± | 0.1 | 1.1 | ± | 0.4 |  |
| tumour | 15.7 | ± | 5.4 | 10.7 | ± | 2.9 | | 19.6 | ± | 6.0 | 9.2 | ± | 5.2 | 6.6 | | ± | 4.8 | 9.8 | ± | 4.6 |  |
| sternum | 1.4 | ± | 0.2 | 1.7 | ± | 0.3 | | 0.9 | ± | 0.2 | 2.2 | ± | 0.6 | 0.2 | | ± | 0.1 | 1.6 | ± | 0.5 |  |
| heart | 3.1 | ± | 0.7 | 2.8 | ± | 0.6 | | 1.4 | ± | 0.3 | 1.3 | ± | 0.6 | 0.1 | | ± | 0.0 | 0.6 | ± | 0.2 |  |
| lung | 3.6 | ± | 1.1 | 2.8 | ± | 0.8 | | 2.0 | ± | 0.3 | 1.8 | ± | 0.4 | 0.2 | | ± | 0.0 | 0.9 | ± | 0.4 |  |
| liver | 13.8 | ± | 1.6 | 17.6 | ± | 6.3 | | 15.6 | ± | 4.4 | 17.8 | ± | 6.8 | 14.2 | | ± | 4.5 | 19.0 | ± | 7.9 |  |
| pancreas | 1.1 | ± | 0.2 | 1.1 | ± | 0.2 | | 0.6 | ± | 0.1 | 0.7 | ± | 0.2 | 0.1 | | ± | 0.0 | 0.4 | ± | 0.2 |  |
| spleen | 2.7 | ± | 0.3 | 3.3 | ± | 0.7 | | 1.9 | ± | 0.3 | 3.0 | ± | 0.9 | 2.0 | | ± | 1.1 | 3.9 | ± | 1.7 |  |
| kidney | 3.0 | ± | 0.6 | 3.0 | ± | 0.7 | | 2.2 | ± | 0.3 | 2.5 | ± | 0.4 | 1.3 | | ± | 0.3 | 2.0 | ± | 0.4 |  |
| muscle | 0.7 | ± | 0.2 | 0.7 | ± | 0.1 | | 0.4 | ± | 0.1 | 0.4 | ± | 0.1 | 0.1 | | ± | 0.1 | 0.2 | ± | 0.1 |  |
| thigh-bone | 0.9 | ± | 0.1 | 1.3 | ± | 0.3 | | 0.6 | ± | 0.1 | 2.2 | ± | 0.7 | 0.4 | | ± | 0.2 | 2.0 | ± | 0.9 |  |
| colon | 1.1 | ± | 0.3 | 1.1 | ± | 0.2 | | 0.7 | ± | 0.2 | 0.6 | ± | 0.2 | 0.2 | | ± | 0.2 | 0.6 | ± | 0.3 |  |
| colon content | 2.3 | ± | 0.8 | 3.9 | ± | 0.8 | | 1.4 | ± | 0.5 | 1.8 | ± | 0.6 | 0.4 | | ± | 0.3 | 0.5 | ± | 0.2 |  |
| ileum | 1.7 | ± | 0.5 | 1.7 | ± | 0.3 | | 0.9 | ± | 0.2 | 0.9 | ± | 0.3 | 0.2 | | ± | 0.2 | 0.7 | ± | 0.5 |  |
| ileum content | 0.5 | ± | 0.1 | 0.5 | ± | 0.2 | | 0.4 | ± | 0.2 | 0.3 | ± | 0.1 | 0.1 | | ± | 0.2 | 0.1 | ± | 0.1 |  |
| stomach | 1.5 | ± | 0.5 | 1.3 | ± | 0.3 | | 0.8 | ± | 0.2 | 0.9 | ± | 0.3 | 0.2 | | ± | 0.2 | 0.4 | ± | 0.2 |  |
| stomach content | 0.1 | ± | 0.1 | 0.2 | ± | 0.2 | | 0.2 | ± | 0.1 | 0.2 | ± | 0.1 | 0.1 | | ± | 0.1 | 0.1 | ± | 0.1 |  |
| brain | 0.3 | ± | 0.1 | 0.2 | ± | 0.1 | | 0.2 | ± | 0.0 | 0.2 | ± | 0.0 | 0.0 | | ± | 0.0 | 0.0 | ± | 0.0 |  |
| head (rest) | 2.0 | ± | 0.4 | 1.7 | ± | 0.5 | | 0.9 | ± | 0.2 | 1.9 | ± | 0.6 | 0.1 | | ± | 0.0 | 1.5 | ± | 0.3 |  |
| knee | 1.3 | ± | 0.2 | 2.5 | ± | 0.6 | | 0.9 | ± | 0.2 | 5.3 | ± | 0.8 | 0.5 | | ± | 0.2 | 5.3 | ± | 1.4 |  |

**Table S4.** Biodistribution of [^89^Zr]Zr-DFO* and [^89^Zr]Zr-DFO, in healthy nude mice, 15 min and 1 h after administration of 1-3 µg of conjugate. Results are expressed as mean (%ID/g) ± sd (n=3 mice per group per time point)

|  | **15 min p.i.** | | | |  |  |  | **1 h p.i.** | | | |  |  |
| --- | --- | --- | --- | --- | --- | --- | --- | --- | --- | --- | --- | --- | --- |
|  | **[^89^Zr]Zr-DFO*** | | | **[^89^Zr]Zr-DFO** | | |  | **[^89^Zr]Zr-DFO*** | | | **[^89^Zr]Zr-DFO** | | |
| blood | 3.2 | ± | 0.8 | 3.4 | ± | 0.9 |  | 0.2 | ± | 0.2 | 0.3 | ± | 0.1 |
| urine | 692.7 | ± | 134.0 | 663.7 | ± | 232.3 |  | 445.8 | ± | 342.3 | 292.1 | ± | 87.2 |
| skin | 3.2 | ± | 0.4 | 3.6 | ± | 1.2 |  | 0.3 | ± | 0.1 | 0.3 | ± | 0.2 |
| tongue | 2.8 | ± | 1.0 | 3.7 | ± | 2.2 |  | 0.2 | ± | 0.1 | 0.2 | ± | 0.1 |
| sternum | 2.8 | ± | 2.3 | 1.8 | ± | 0.9 |  | 0.2 | ± | 0.1 | 0.1 | ± | 0.1 |
| heart | 1.3 | ± | 0.3 | 1.5 | ± | 0.3 |  | 0.1 | ± | 0.1 | 0.1 | ± | 0.1 |
| lung | 2.4 | ± | 0.6 | 2.6 | ± | 0.5 |  | 0.3 | ± | 0.1 | 0.4 | ± | 0.2 |
| liver | 0.8 | ± | 0.2 | 1.3 | ± | 0.3 |  | 0.1 | ± | 0.1 | 0.6 | ± | 0.2 |
| spleen | 0.8 | ± | 0.1 | 0.9 | ± | 0.2 |  | 0.1 | ± | 0.0 | 0.1 | ± | 0.1 |
| kidney | 23.6 | ± | 12.1 | 34.7 | ± | 13.2 |  | 3.4 | ± | 0.7 | 5.4 | ± | 1.3 |
| bladder | 10.8 | ± | 6.9 | 10.8 | ± | 5.5 |  | 9.9 | ± | 10.0 | 1.5 | ± | 1.1 |
| muscle | 0.9 | ± | 0.4 | 0.8 | ± | 0.2 |  | 0.3 | ± | 0.4 | 0.1 | ± | 0.1 |
| thigh-bone | 0.7 | ± | 0.3 | 0.9 | ± | 0.2 |  | 0.1 | ± | 0.1 | 0.2 | ± | 0.2 |
| colon | 1.6 | ± | 0.4 | 1.2 | ± | 0.2 |  | 0.2 | ± | 0.2 | 0.1 | ± | 0.1 |
| colon content | 0.1 | ± | 0.1 | 0.2 | ± | 0.2 |  | 0.1 | ± | 0.1 | 0.1 | ± | 0.0 |
| ileum | 1.1 | ± | 0.3 | 0.7 | ± | 0.3 |  | 0.1 | ± | 0.1 | 0.1 | ± | 0.1 |
| ileum content | 1.2 | ± | 0.4 | 0.9 | ± | 0.2 |  | 0.1 | ± | 0.1 | 0.4 | ± | 0.3 |
| stomach | 1.0 | ± | 0.2 | 1.1 | ± | 0.1 |  | 0.4 | ± | 0.5 | 0.1 | ± | 0.1 |
| stomach content | 0.0 | ± | 0.0 | 0.4 | ± | 0.5 |  | 0.1 | ± | 0.1 | 0.1 | ± | 0.1 |
| head | 1.7 | ± | 0.5 | 1.4 | ± | 0.3 |  | 0.2 | ± | 0.1 | 0.2 | ± | 0.1 |
| knee | 1.6 | ± | 0.4 | 1.5 | ± | 0.5 |  | 0.1 | ± | 0.1 | 0.1 | ± | 0.1 |

**Table S5.** Biodistribution of [^89^Zr]Zr-DFO*-NCS-trastuzumab, [^89^Zr]Zr-DFO-NCS-trastuzumab, [^89^Zr]Zr-DFO*-NCS-B12 and [^89^Zr]Zr-DFO-NCS-B12, in a BT-474 intratibial model of metastasis in nude mice, 144 h after administration of 100 µg of conjugate. Results are expressed as mean (%ID/g) ± sd (n=5-6 mice per group)

| **144h** | **[^89^Zr]Zr-DFO*-NCS-trastuzumab** | | | | | **[^89^Zr]Zr-DFO-NCS-trastuzumab** | | | | **[^89^Zr]Zr-DFO*-NCS-B12** | | | | **[^89^Zr]Zr-DFO-NCS-B12** | | |  |
| --- | --- | --- | --- | --- | --- | --- | --- | --- | --- | --- | --- | --- | --- | --- | --- | --- | --- |
| blood | | | 8.5 | ± | 1.2 | 7.5 | | ± | 1.7 | 7.2 | | ± | 2.0 |  | 5.4 | ± | 1.4 |
| urine | | | 2.4 | ± | 0.6 | 1.1 | | ± | 1.1 | 1.4 | | ± | 0.9 |  | 0.7 | ± | 0.3 |
| skin | | | 3.8 | ± | 0.6 | 4.3 | | ± | 1.2 | 3.6 | | ± | 0.7 |  | 4.0 | ± | 0.6 |
| bladder | | | 3.3 | ± | 0.6 | 3.5 | | ± | 0.6 | 3.5 | | ± | 0.8 |  | 3.3 | ± | 0.5 |
| sternum | | | 1.7 | ± | 0.3 | 3.1 | | ± | 0.9 | 1.5 | | ± | 0.2 |  | 3.4 | ± | 0.7 |
| heart | | | 2.5 | ± | 0.1 | 2.6 | | ± | 0.5 | 2.3 | | ± | 0.5 |  | 1.9 | ± | 0.3 |
| lung | | | 4.3 | ± | 1.0 | 3.7 | | ± | 1.0 | 3.6 | | ± | 0.9 |  | 3.5 | ± | 0.4 |
| liver | | | 4.5 | ± | 1.2 | 5.6 | | ± | 2.2 | 4.7 | | ± | 1.4 |  | 8.9 | ± | 2.7 |
| pancreas | | | 1.0 | ± | 0.2 | 0.9 | | ± | 0.3 | 0.9 | | ± | 0.2 |  | 0.9 | ± | 0.1 |
| spleen | | | 2.9 | ± | 0.6 | 4.3 | | ± | 1.1 | 4.0 | | ± | 1.4 |  | 4.1 | ± | 1.2 |
| kidney | | | 3.1 | ± | 0.3 | 5.9 | | ± | 4.1 | 4.3 | | ± | 0.4 |  | 5.2 | ± | 0.7 |
| muscle | | | 0.8 | ± | 0.1 | 0.7 | | ± | 0.1 | 0.6 | | ± | 0.1 |  | 0.5 | ± | 0.1 |
| thigh-bone left | | | 1.4 | ± | 0.4 | 4.3 | | ± | 1.4 | 1.6 | | ± | 0.5 |  | 5.2 | ± | 2.0 |
| tumour tibia | | | 10.2 | ± | 4.2 | 15.1 | | ± | 4.0 | 1.8 | | ± | 0.3 |  | 7.1 | ± | 2.6 |
| thigh-bone right | | | 1.6 | ± | 0.4 | 3.8 | | ± | 1.9 | 1.6 | | ± | 0.5 |  | 5.5 | ± | 1.2 |
| unaffected tibia | | | 1.6 | ± | 0.2 | 4.7 | | ± | 1.3 | 1.5 | | ± | 0.3 |  | 5.7 | ± | 1.4 |
| colon | | | 1.0 | ± | 0.2 | 1.6 | | ± | 0.5 | 1.2 | | ± | 0.2 |  | 1.3 | ± | 0.5 |
| colon content | | | 0.9 | ± | 0.3 | 0.9 | | ± | 0.2 | 1.7 | | ± | 0.4 |  | 1.5 | ± | 0.2 |
| ileum | | | 1.7 | ± | 0.4 | 2.2 | | ± | 0.9 | 1.6 | | ± | 0.4 |  | 1.8 | ± | 0.8 |
| ileum content | | | 0.6 | ± | 0.4 | 0.8 | | ± | 0.3 | 0.6 | | ± | 0.2 |  | 0.6 | ± | 0.2 |
| stomach | | | 1.1 | ± | 0.1 | 1.2 | | ± | 0.3 | 1.2 | | ± | 0.2 |  | 1.2 | ± | 0.4 |
| stomach content | | | 0.3 | ± | 0.0 | 0.2 | | ± | 0.1 | 0.2 | | ± | 0.2 |  | 0.3 | ± | 0.1 |
| brain | | | 0.3 | ± | 0.0 | 0.3 | | ± | 0.1 | 0.3 | | ± | 0.1 |  | 0.2 | ± | 0.0 |
| head (rest) | | | 1.7 | ± | 0.5 | 2.3 | | ± | 0.7 | 1.3 | | ± | 0.5 |  | 2.4 | ± | 0.3 |

**Table S6.** Biodistribution of [^89^Zr]Zr-oxalate (1 MBq, n= 3 mice), [^89^Zr]Zr-citrate (0.5 MBq, n= 4 mice) and [^89^Zr]Zr-chloride (0.5 MBq, n=4 mice), in a BT-474 intratibial model of metastasis in nude mice, 24 h after administration. Results are expressed as mean (%ID/g) ± sd. *In early development of the BT-474 bone model, dislocation of the thigh-bones for tissue collection in the oxalate group was performed differently than later in the citrate and chloride groups explaining the differences in %ID/g of tissue. PET imaging (Figure S3) confirmed the absence of difference between the groups

| **24 h p.i.** | **[^89^Zr]Zr-oxalate** | | | **[^89^Zr]Zr-citrate** | | | **[^89^Zr]Zr-chloride** | | |
| --- | --- | --- | --- | --- | --- | --- | --- | --- | --- |
| blood | 4.5 | ± | 0.5 | 4.1 | ± | 1.5 | 4.3 | ± | 1.2 |
| urine | 2.1 | ± | 0.1 | 2.7 | ± | 2.4 | 1.8 | ± | 0.1 |
| skin | 3.9 | ± | 0.1 | 3.1 | ± | 0.8 | 3.5 | ± | 0.5 |
| bladder | 2.2 | ± | 0.1 | 1.9 | ± | 0.6 | 2.4 | ± | 0.7 |
| sternum | 8.8 | ± | 0.7 | 8.3 | ± | 1.4 | 9.4 | ± | 2.5 |
| heart | 2.0 | ± | 0.4 | 1.7 | ± | 0.7 | 1.7 | ± | 0.5 |
| lung | 3.1 | ± | 0.1 | 2.1 | ± | 1.0 | 3.1 | ± | 0.9 |
| liver | 3.3 | ± | 0.4 | 4.4 | ± | 1.0 | 3.3 | ± | 0.7 |
| pancreas | 1.2 | ± | 0.0 | 1.1 | ± | 0.2 | 1.2 | ± | 0.2 |
| spleen | 1.8 | ± | 0.0 | 4.8 | ± | 1.1 | 1.9 | ± | 0.4 |
| kidney | 4.5 | ± | 0.1 | 3.9 | ± | 0.8 | 4.0 | ± | 0.5 |
| muscle | 0.8 | ± | 0.1 | 0.6 | ± | 0.2 | 0.7 | ± | 0.2 |
| thigh-bone left | 8.2* | ± | 0.1 | 16.2 | ± | 3.4 | 18.3 | ± | 3.3 |
| tumour tibia | 17.0 | ± | 4.4 | 16.8 | ± | 6.0 | 18.1 | ± | 4.0 |
| thigh-bone right | 8.5* | ± | 0.7 | 17.3 | ± | 4.3 | 17.8 | ± | 2.3 |
| unaffected tibia | 13.3 | ± | 0.4 | 16.4 | ± | 4.1 | 16.6 | ± | 3.5 |
| colon | 1.2 | ± | 0.0 | 1.1 | ± | 0.1 | 1.2 | ± | 0.3 |
| colon content | 1.4 | ± | 0.1 | 2.1 | ± | 2.0 | 0.9 | ± | 0.7 |
| ileum | 1.2 | ± | 0.0 | 1.3 | ± | 0.6 | 1.7 | ± | 0.2 |
| ileum content | 0.9 | ± | 0.3 | 0.7 | ± | 0.4 | 1.0 | ± | 0.4 |
| stomach | 1.0 | ± | 0.2 | 1.1 | ± | 0.2 | 1.2 | ± | 0.3 |
| stomach content | 0.1 | ± | 0.0 | 0.4 | ± | 0.3 | 0.2 | ± | 0.1 |
| brain | 0.1 | ± | 0.0 | 0.1 | ± | 0.1 | 0.2 | ± | 0.0 |
| head (rest) | 3.8 | ± | 0.8 | 5.1 | ± | 0.5 | 4.8 | ± | 0.7 |


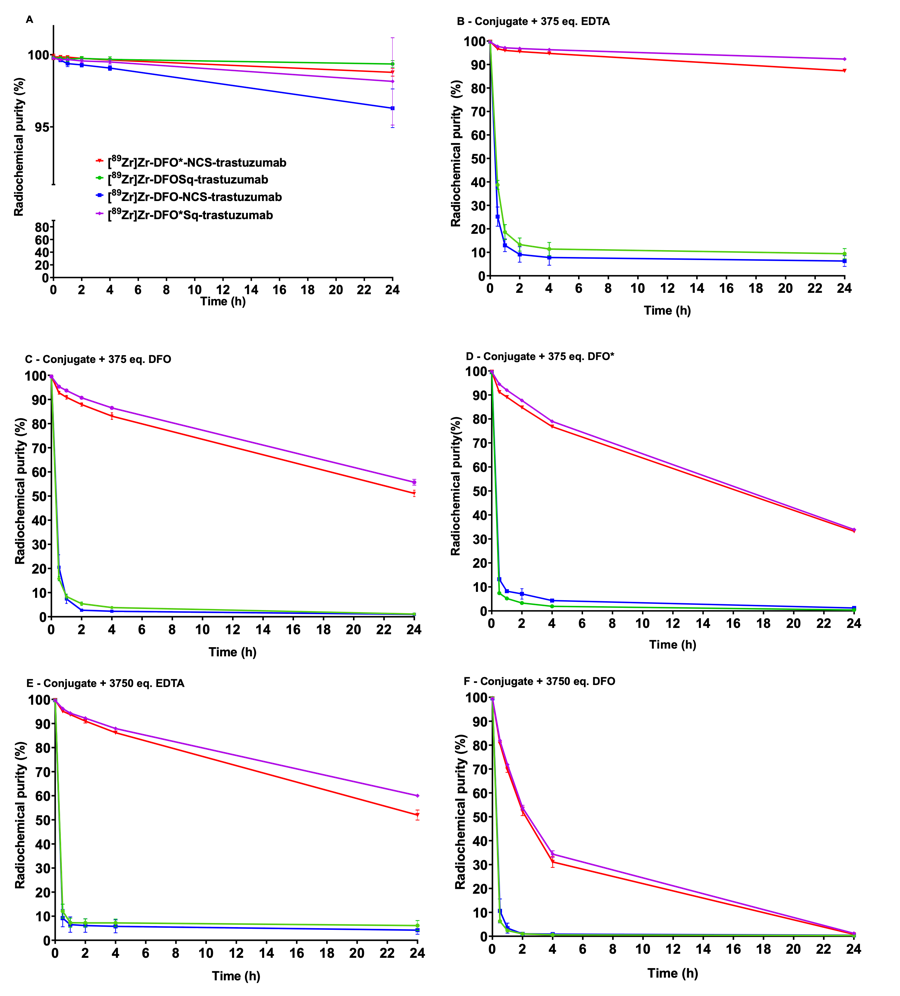


**Figure S1**. Stability of [^89^Zr]Zr-DFO*-NCS-trastuzumab, [^89^Zr]Zr-DFOSq-trastuzumab, [^89^Zr]Zr-DFO-NCS-trastuzumab, and [^89^Zr]Zr-DFO*Sq-trastuzumab incubated for 24 h at 37ºC, at pH 5.5 **(A)** and when challenged with either 375 equivalents of EDTA **(B),** DFO **(C)** and DFO* **(D)** or 3750 equivalents of EDTA **(E)** and DFO **(F)**.


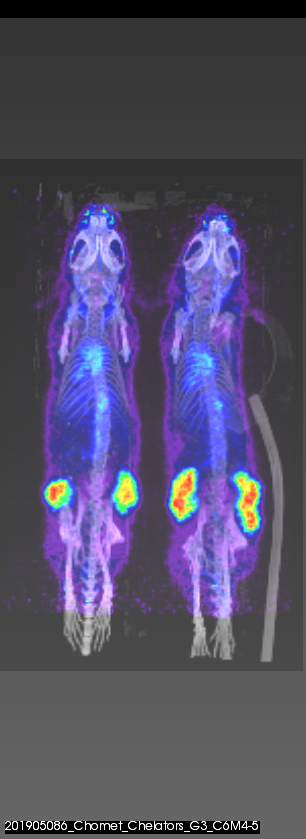

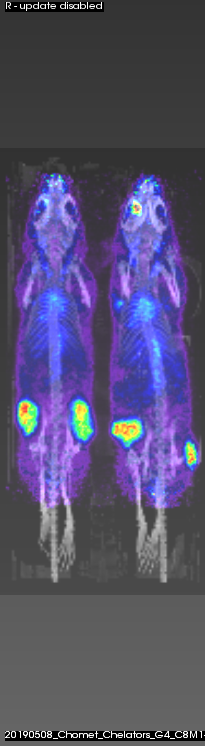

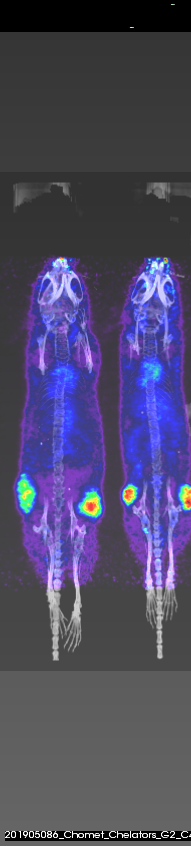

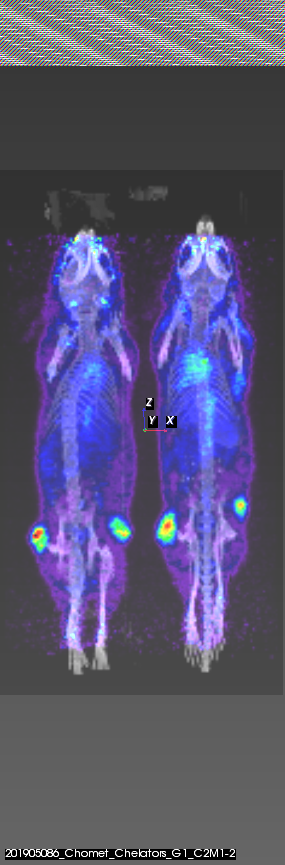


**DFO*-NCS DFOSq DFO-NCS DFO*Sq**

**24 h**

**41 %ID/g**

**0**

**A B C D**


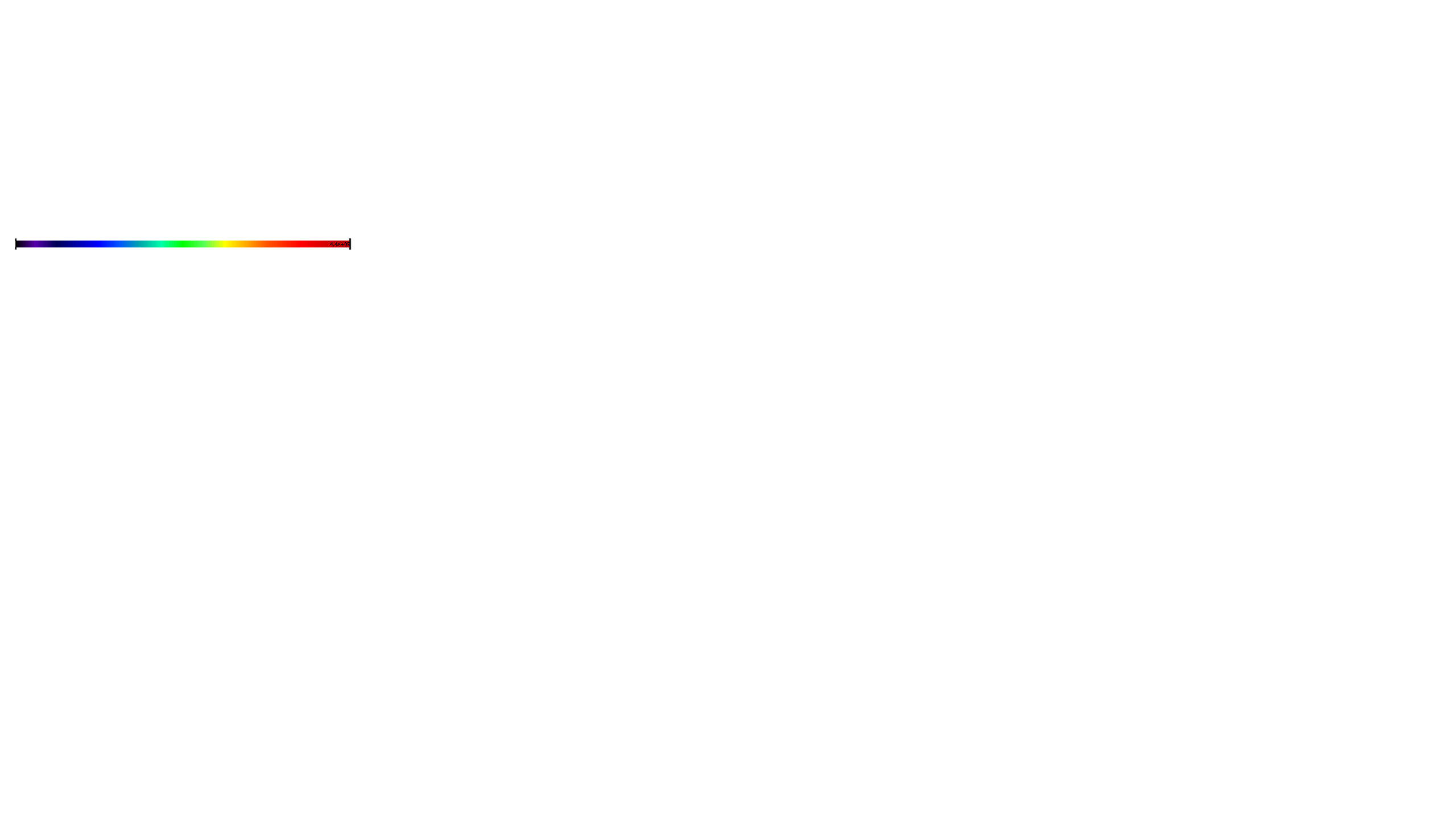

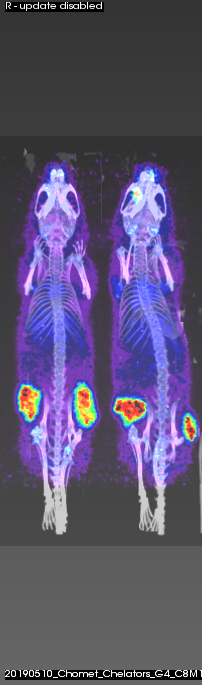

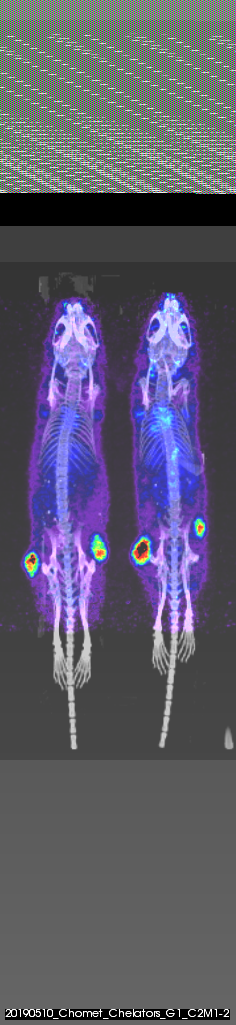

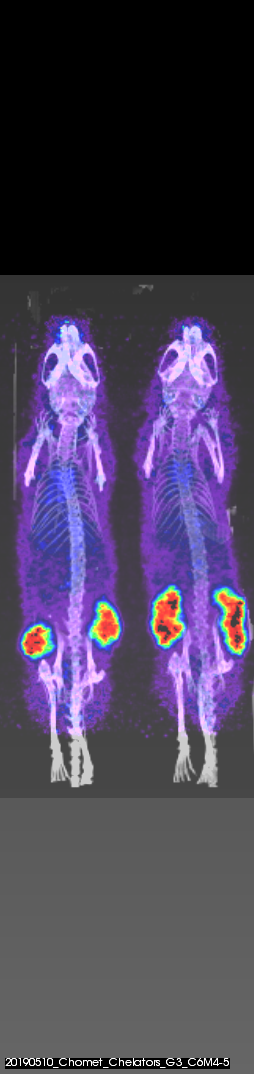

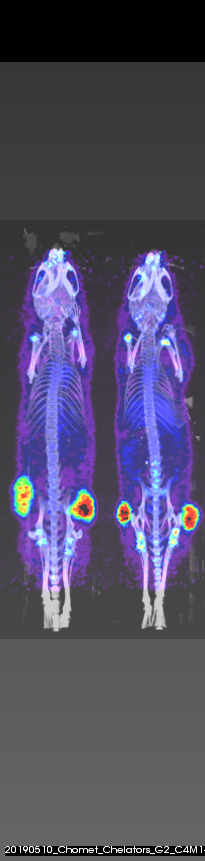


**DFO*-NCS DFOSq DFO-NCS DFO*Sq**

**72 h**

**41 %ID/g**

**0**

**A B C D**


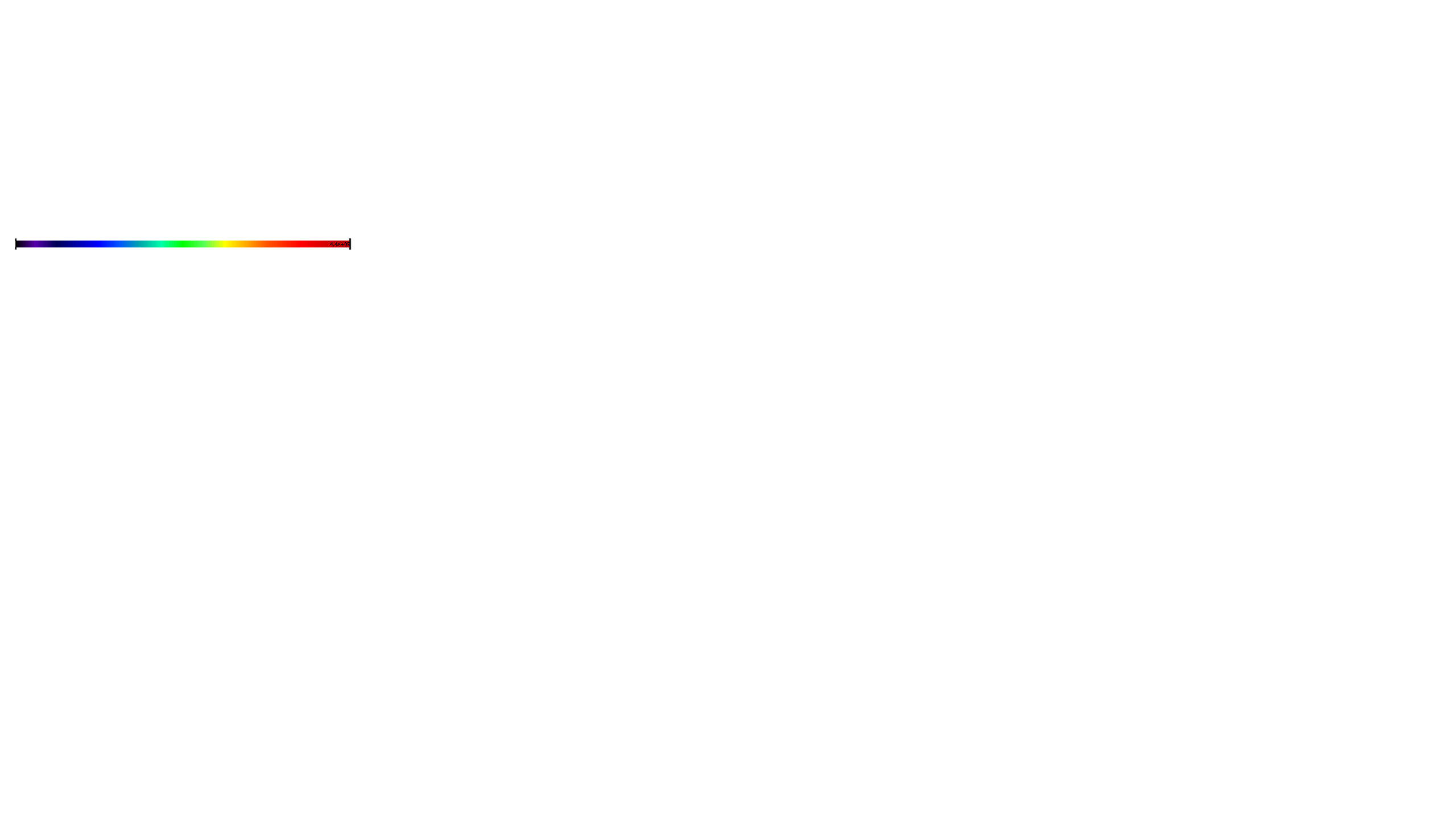


**Figure S2.** PET images of N87 tumour bearing mice injected with 110 µg of either [^89^Zr]Zr-DFO*-NCS-trastuzumab **(A)**, [^89^Zr]Zr-DFOSq-trastuzumab **(B)**, [^89^Zr]Zr-DFO-NCS-trastuzumab **(C)**, or [^89^Zr]Zr-DFO*Sq-trastuzumab **(D)** and scanned 24 and 72 h p.i. Images are presented as Maximum Intensity Projections (MIP). Tumours are indicated with red arrows and bone uptake with blue arrows


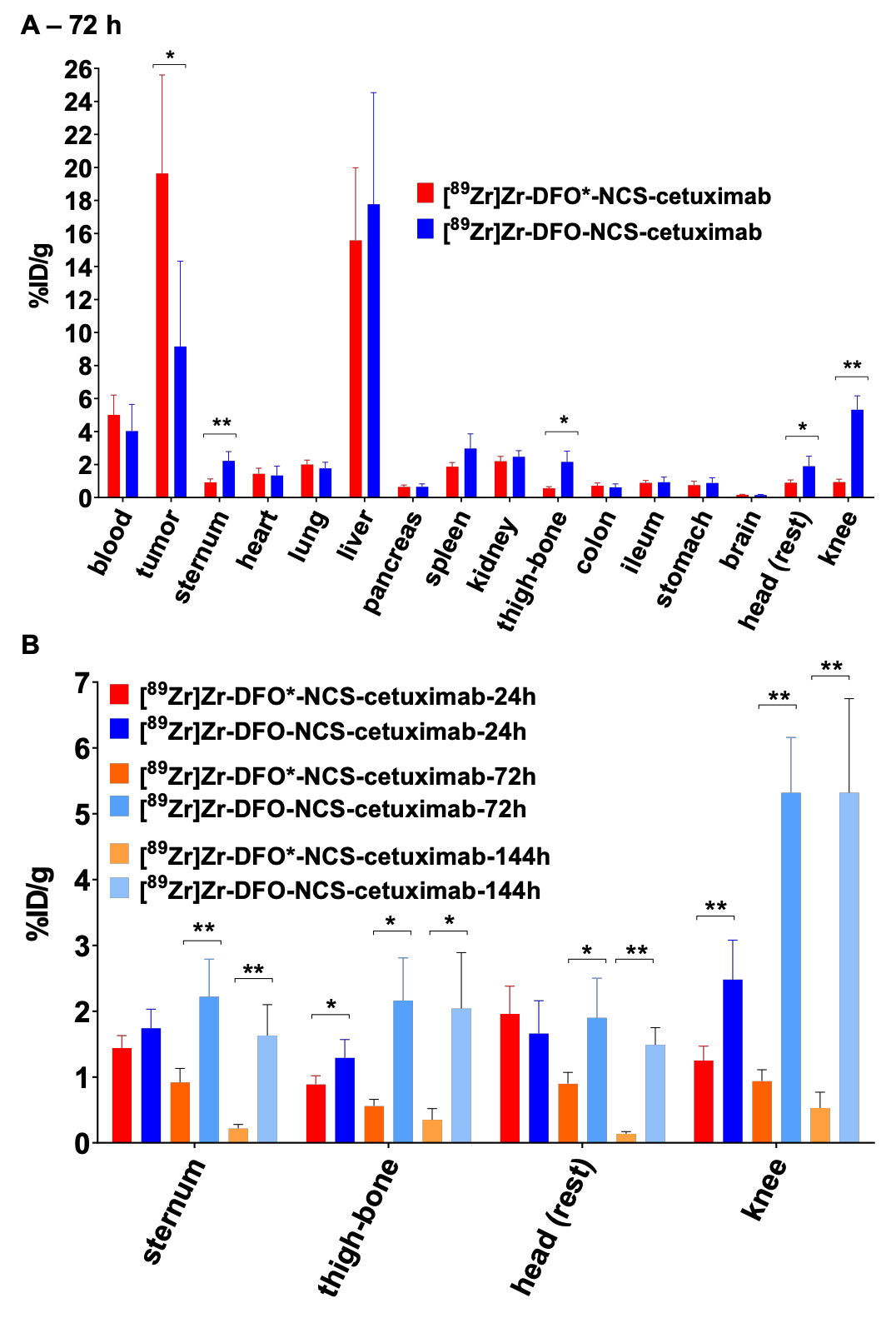


**Figure S3**. Biodistribution of [^89^Zr]Zr-DFO*-NCS-cetuximab and [^89^Zr]Zr-DFO-NCS-cetuximab at 72 h p.i. **(A)** and in collected bone containing organs in A431 tumour-bearing nude mice at 24, 72 and 144 h p.i. **(B)** of 100 µg of the respective conjugates. Uptake expressed as %ID/g (Mean ±SD, n=5 animals per group). Significant differences between DFO*-NCS-cetuximab and DFO-NCS-cetuximab compared at the same time point are marked with asterisks (* p<0.05, ** p<0.01)

**
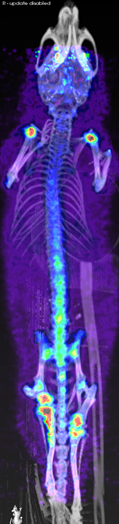

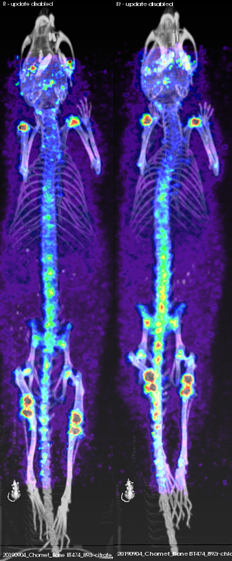
**

**A B**  **C**

**61 %ID/g**

**0**


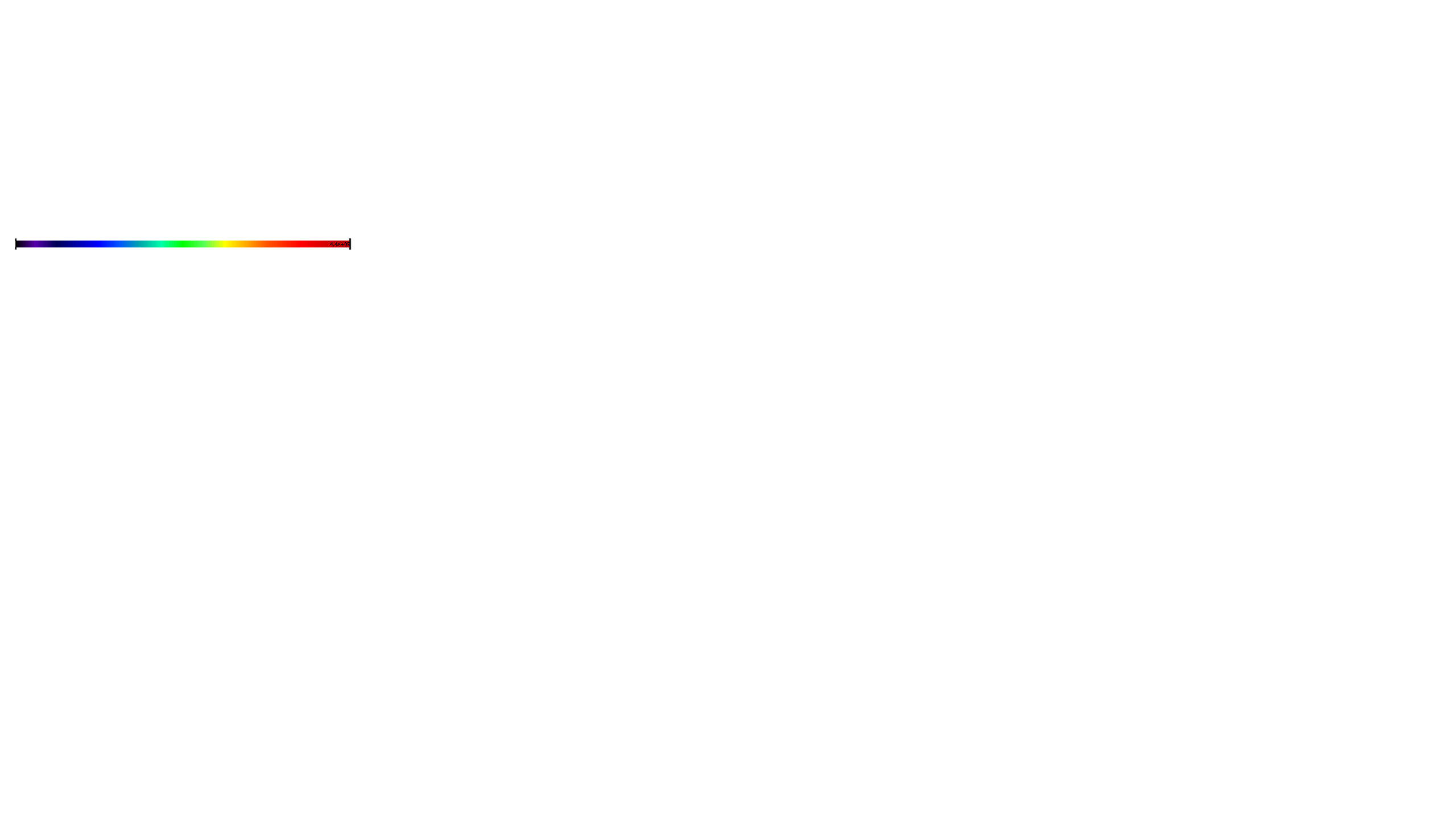


**Figure S4.** PET images of mice injected with either [^89^Zr]Zr-oxalate **(A)**, [^89^Zr]Zr-citrate **(B)** or [^89^Zr]Zr-chloride **(C)** and scanned 24 h p.i. All mice had received an intratibial injection of BT-474 cells in the left leg and PBS in the right leg. Images are presented as Maximum Intensity Projections (MIP).

**References**

1. Vugts DJ, Klaver C, Sewing C, Poot AJ, Adamzek K, Huegli S, et al. Comparison of the octadentate bifunctional chelator DFO*-pPhe-NCS and the clinically used hexadentate bifunctional chelator DFO-pPhe-NCS for 89Zr-immuno-PET. Eur J Nucl Med Mol Imaging. 2017;44:286–95.

2. Vosjan MJWD, Perk LR, Visser GWM, Budde M, Jurek P, Kiefer GE, et al. Conjugation and radiolabeling of monoclonal antibodies with zirconium-89 for PET imaging using the bifunctional chelate p-isothiocyanatobenzyl-desferrioxamine. Nat Protoc. 2010;5:739–43.

3. Rudd SE, Roselt P, Cullinane C, Hicks RJ, Donnelly PS. A desferrioxamine B squaramide ester for the incorporation of zirconium-89 into antibodies. Chem Commun. The Royal Society of Chemistry; 2016;52:11889–92.

4. Pandya DN, Bhatt NB, Almaguel F, Rideout-Danner S, Gage HD, Solingapuram Sai KK, et al. Zirconium-89 chloride can be used for immuno-PET radiochemistry without loss of antigen reactivity in vivo. J Nucl Med. 2019;60:696–701.

5. Lindmo T, Boven E, Cuttitta F, Fedorko J, Bunn PA. Determination of the immunoreactive function of radiolabeled monoclonal antibodies by linear extrapolation to binding at infinite antigen excess. J Immunol Methods. 1984;72:77–89.

6. Sijbrandi NJ, Merkul E, Muns JA, Waalboer DCJ, Adamzek K, Bolijn M, et al. A Novel Platinum(II)-Based Bifunctional ADC Linker Benchmarked Using Zr-Desferal and Auristatin F Conjugated Trastuzumab. Cancer Res. 2017;77:257–67.

7. Pandya DN, Bhatt N, Yuan H, Day CS, Ehrmann BM, Wright M, et al. Zirconium tetraazamacrocycle complexes display extraordinary stability and provide a new strategy for zirconium-89-based radiopharmaceutical development. Chem Sci. Royal Society of Chemistry; 2017;8:2309–14.

8. Deri MA, Ponnala S, Zeglis BM, Pohl G, Dannenberg JJ, Lewis JS, et al. Alternative chelator for ^89^Zr radiopharmaceuticals: radiolabeling and evaluation of 3,4,3-(LI-1,2-HOPO). J Med Chem. American Chemical Society; 2014;57:4849–60.
